# Speed-accuracy trade-offs in action perception, motor imagery, and execution of hand movements in autistic and non-autistic adults

**DOI:** 10.1038/s41598-025-97036-w

**Published:** 2025-04-17

**Authors:** Ying Bai, Molly Brillinger, April Karlinsky, Ellen Poliakoff, Timothy N. Welsh, Emma Gowen

**Affiliations:** 1 Division of Psychology Communication and Human Neuroscience, The University of Manchester, Manchester, UK; 2 Faculty of Kinesiology & Physical Education, University of Toronto, Toronto, ON Canada; 3 Department of Kinesiology, California State University-San Bernardino, San Bernardino, CA USA

**Keywords:** Autism, Motor control, Perception-action coupling, Explicit motor imagery, Action perception, Cognitive neuroscience, Neuroscience, Sensory processing, Autism spectrum disorders

## Abstract

Action perception, execution, and imagery share motor-cognitive processes. Given prevalent sensory and motor coordination difficulties in autism, the processes of action perception and imagery may also be altered. This study investigated whether autistic adults can engage in motor imagery by testing potential differences in executing, perceiving, and imagining hand movements between autistic and non-autistic adults. Twenty autistic individuals and twenty age- and IQ-matched controls completed execution, imagination, and perception tasks using a Fitts’ Law paradigm in an online session. For the execution and imagination tasks, participants performed or imagined making aiming movements between two targets. For the action perception task, participants indicated whether they could perform as accurately as the movements in presented videos. Target size and distance were manipulated into three difficulty levels and systematically varied across all tasks. Results showed Fitts’ Law relationships for all tasks for both groups, with significant positive correlations between movement times and difficulty level. Movement times were longest in the imagination task and shortest in the perception task for both groups. These findings suggest motor imagery processes are relatively intact in autistic adults, highlighting that further investigation of motor imagery as a therapy for motor coordination difficulties in autistic individuals is warranted.

**Supplementary Information:**

The online version contains supplementary material available at 10.1038/s41598-025-97036-w.

## Introduction

Autism Spectrum Condition (ASC) is a neurodevelopmental disorder characterized by difficulties in communication and social interaction, as well as restricted interests and repetitive behaviours^1^. Alongside these challenges, approximately 80% of autistic individuals experience altered motor coordination, including difficulties with fine motor control, hand-eye coordination, balance, and gait^2–6^. Persisting from infancy to adulthood, these motor challenges can have a negative impact on autistic individuals’ lives by reducing their skills to carry out daily tasks such as navigating cluttered environments, preparing food, getting dressed, or tying shoelaces^4^. Such motor challenges can also produce anxiety, fear of judgment, and frustration and reduce social opportunities through exclusion by others or self-exclusion^4^.

Motor coordination difficulties may also impact social skills through motor-cognitive mechanisms associated with action simulation. It has been proposed that when an individual observes another person, they internally simulate the other person’s actions by activating their own motor, cognitive, and emotional representations^7,8^. Simulation provides a basic framework in which motor processes can influence various aspects of social interaction, including action perception (e.g., action understanding), imitation, theory of mind, empathy, and language^7,9–11^. Altered motor coordination may impact upon simulation capability affecting these social processes because simulation processes are thought to rely on the same neural networks and perception-action representations that enable motor execution. Thus, if motor coordination challenges are associated with weak or disrupted perception-action codes, then simulation (and resulting action perception and imagery) that rely on those same codes will also be challenged.

To elucidate, action perception and execution are thought to be linked because these abilities rely on a shared set of motor-cognitive processes^8,12^and integrated perception-action coding systems. According to Ideomotor Coding or Common Coding Theories^13–15^, perception and motor systems store and transfer abstract information in linked neural codes and networks. Specifically, it is suggested that the codes representing the perceptual consequences of an action (e.g., a letter appearing on the computer screen) are tightly linked or coupled to the motor codes representing the muscle contractions that would bring about an action (e.g., the flexion of a finger over a specific key). It is further proposed that the internal activation of one of the perception (or action) codes necessarily activates the linked action (or perception) codes. Research testing predictions of such ideomotor theories has generally provided evidence consistent with these predictions. For example, in support of the prediction that action perception activates motor codes, numerous studies have shown that observing another individual’s actions can influence the observer’s own movements, either enhancing imitation or interfering with planned actions^16–19^. These facilitation or interference effects emerge because the perception-evoked response codes are compatible or incompatible with the goal actions, respectively. Hardwick et al.^20^ conducted a meta-analysis and identified a common brain network, including premotor, parietal, and somatosensory areas, that are activated during various motor-related tasks, such as action observation, movement execution, and motor imagery (imagining movement; described in greater detail below).

Because of the potential links between action execution, action perception, and social interactions, investigating possible differences between autistic and non-autistic individuals in these abilities is an active area of autism research. Historically, action perception and imitation has been a primary focus of many studies (likely because of the more direct associations between these processes and social interactions). In regards to action perception, compared with non-autistic individuals, autistic participants generally demonstrate proficiency in simpler tasks, such as the detection of human motion or direction of motion^21–24^. However, they tend to have more challenges with complex tasks that demand a deeper understanding of social cues, especially emotions^25–30^. Altered imitation is also observed with autistic individuals showing reduced replication of the kinematic style of observed actions^31–34^.

A separate motor-cognitive behaviour that might be related to action execution and perception through perception-action coding is motor imagery (MI). MI is a specialized form of mental simulation that occurs when an individual imagines performing an action without physically executing that action^35^. Like action perception, MI is thought to occur via simulation involving the sub-threshold activation of perception-action codes for a specific movement^12,36,37^. Indeed, there is a substantial literature demonstrating that MI involves activation of the motor system. MI can be performed in the visual or kinesthetic modality with the former involving the visualisation of an action and the latter consisting of imagining the sensations of the action^38^. Jeannerod^38^ introduced the concept of explicit and implicit imagery, differentiated by the degree of cognitive involvement during the task. Explicit imagery tasks require participants to consciously engage in imagining movements and subsequently report or rate their imagery experience (e.g., mental chronometry tasks or questionnaires), whereas implicit imagery tasks do not necessitate conscious engagement in the imagery process (e.g., hand rotation task).

A recent systematic review investigated MI in autism and found evidence that autistic individuals can use implicit MI, while research on explicit MI remains insufficient^4^. Explicit imagery is often tested using questionnaires. The Kinesthetic and Visual Imagery Questionnaire (KVIQ) is a widely used tool in which participants are asked to perform simple movements, then to imagine themselves executing the same movements, and then to rate the clarity of their images (visual subscale) and intensity of their sensations (kinesthetic subscale) on a 5-point scale (1 – no image/no sensations to 5 – very clear image/very intensive sensations)^39,40^. Only one study has used this approach with autistic individuals, finding no group differences between autistic and non-autistic adults on either subscale of the KVIQ^41^. In contrast, other explicit imagery studies that have asked autistic adults to imagine themselves performing a spatial bimanual task^42 ^or recall a series of actions following imagining performing those actions^43 ^have shown explicit MI to be absent in the autistic group. However, it remains uncertain whether this lack of explicit MI reflects an inherent characteristic or a strategic choice by autistic participants to abstain from using imagery^44^.

Another approach to assessing explicit MI is mental chronometry^45^. This method involves comparisons of the movement times (MTs) of participants who have executed and imagined the same action task. The (in)consistencies of MTs between the tasks are used to infer the properties of MI – the closer the imagined MTs are to executed MTs the more accurate the simulation. It is posited that the time taken to actually execute versus imagine the same movement should be similar, otherwise an impairment of motor imagery may be indicated^46^. Mental chronometry has yet to be explored in the context of autism, yet it is a useful approach to examining MI.

The aim of the current study was to develop a deeper understanding of the potentially linked abilities of action execution, perception, and imagination in autistic and non-autistic people using mental chronometry. The Fitts’ Law reciprocal aiming task is a well-established behavioural task for investigating the relationships among action execution, perception, and imagination^47,48,49^. In this task, participants are asked to move a stylus or their finger between two targets as quickly as possible while maintaining accuracy^50^. Both the width of the targets and the distance between them are manipulated to create varying levels of movement difficulty, referred to as the Index of Difficulty (ID). According to Fitts’ Law, as task difficulty increases, MT should increase to maintain accuracy, reflecting a speed-accuracy trade-off. Evidence supports the applicability of Fitts’ Law across execution, perception, and imagination tasks^47,46,51–54^. For example, Wong et al.^46 ^ found that relationships between MT and ID did not differ in non-autistic individuals across execution, perception, and imagination. Further, the consistency between MTs across action execution, perception, and imagination increased after the participants gained experience executing the movements. These findings are consistent with predictions based on common coding accounts of these abilities. However, imagined MTs were longer than both executed and perceived MTs, which did not differ. This pattern of results suggests that additional cognitive resources are required to imagine movements due to the mental effort of maintaining the active imagination^55^.

In summary, it remains unclear whether autistic individuals can perform explicit MI, and how such MI processes relate to action execution and perception. Therefore, the present research study aimed to investigate mental chronometry of a movement execution task for the first time in autistic individuals using a Fitts’ Law paradigm to examine whether there are differences in executing, perceiving, and imagining actions between autistic and non-autistic adults. Previous studies examining execution in the Fitts’ aiming tasks in autistic individuals have shown the existence of a relationship between task difficulty and executed MTs^56^. First, we predicted that if simulation processes are present and intact in autistic individuals, a positive relationship between MT and ID would emerge across the perception and imagination tasks. Second, if simulation processes are affected in the autistic group, these individuals may show lower correlation coefficients between MT and ID in the perception and imagery tasks than the non-autistic group. Third, if simulation processes are affected in the autistic group, they may show a significantly greater difference in MTs between execution, perception, and imagination tasks compared to the non-autistic group.

## Materials and methods

This study was pre-registered using the open science framework: https://osf.io/26azs/?view_only=80cb8ea8c42144e0b95fc03842189583.

### Participants

A total of 20 autistic and 20 non-autistic participants of similar age, sex, handedness and full-scale intelligence quotient (IQ) were recruited through the laboratory database, the Autism@Manchester mailing list, local support groups and volunteer advertisements (see Table 1 for participant demographics). Because there were no data from previous studies examining the imagination of a Fitts’ Law task in autistic participants, sample size was based on an earlier study demonstrating Fitts’ Law relationships in action execution, perception, and imagination among 20 non-autistic participants^46^. Specifically, the group mean and standard deviations for each ID and task (action execution, perception, and imagination) from Wong et al.^46^ were used to create data for 20 participants across 10,000 simulated experiments in R (rnorm function, R version 3.5.3). An ID x Task repeated measures ANOVA (r function aov4) was run on each of these experiments and the number of times the *p*-value fell below 0.05 was calculated. Power was > 0.99 for the main effects of Task and ID and > 0.1 for the interaction. R packages for the simulation included tidyverse, dplyr, broom, and afex. A sensitivity analyses used G*Power were conduct based on the observed effect sizes from our mixed ANOVA model (see Supplementary materials).

All autistic participants had received a professional diagnosis of autism. Autistic participants also scored above the cut-off for autism (≥ 60) on the Social Responsiveness Scale (SRS- 2)^57^. None of the participants from either group reported any psychological or neurological disorders (e.g., Parkinson’s) or learning disabilities, and all reported normal or corrected-to-normal vision. The autistic group scored higher than the non-autistic group on both the SRS- 2, the Adult Developmental Coordination Disorders/Dyspraxia Checklist (ADC)^58^, but scored lower on both the Visual imagery and Kinesthetic imagery subscales on the Kinesthetic and Visual Imagery Questionnaire (KVIQ)^39^ (Table 1; see also Methods section for additional details). These scales were used to characterize the sample and assess their relation to MI performance in the task. All participants were provided informed consent during the study. Consent was collected via an online survey platform link (Qualtrics), and the study was approved by the University of Manchester Research Ethics Committee (Review Reference: 2021–11748- 20016). All experiments were performed in accordance with the University’s Code of Good Research Conduct.

**Table 1:** Participant demographics.

	Autistic (*n* = 20)	Non-autistic (*n* = 20)	Group comparison
Age	29.30 ± 7.66	28.95 ± 6.44	*t* (38) = 0.16, *p* = 0.88
Sex	5 Male, 15 Female	7 Male, 13 Female	*X*^2^ (1, *N* = 40) = 0.48, *p* = 0.49
^†^EHI	17 right-handed	19 right-handed	*X*^2^ (1, *N* = 40) = 0.28, *p* = 0.60
FSIQ- 2	120.75 ± 13.74	113.00 ± 14.56	*t* (38) = 1.73, *p* = 0.09
KVIQ^*^	VI: 14.90 ± 6.33 KI: 10.95 ± 3.83	VI: 19.45 ± 4.21 KI: 16.35 ± 3.70	*t* (38) = − 2.68, *p =* 0.01 *t* (38) = − 4.53, *p* < 0.001
SRS- 2^*^	110.95 ± 21.57	44.50 ± 15.65	*t* (38) = 11.15, *p* < 0.001
ADC^*^	93.10 ± 14.82	65.20 ± 10.36	*t* (38) = 6.90, *p* < 0.001

EHI: Edinburgh Handedness Inventory, FSIQ- 2: Full scale IQ with 2 subtest scores (Matrix Reasoning and Vocabulary), KVIQ: Kinesthetic and Visual Imagery Questionnaire (VI: Visual Imagery, KI: Kinesthetic Imagery) which assesses participants’ ability to maintain motor imagery, SRS- 2: Social Responsiveness Scale, Second Edition which is assess social communication difficulties, ADC: Adult Developmental Coordination Disorders/Dyspraxia Checklist which is evaluate motor coordination difficulties. Mean is shown with ± standard deviation. “*” indicates statistically significant group difference. ^†^9 participants (5 from the autistic group and 4 from the non-autistic group) were mixed-handed. Of these, 8 used their right hand as their preferred hand to complete the tasks, while 1 autistic participant used their left hand. In the final analysis, we reported the hand that was actually used for the tasks.

## Apparatus, stimuli and task

The design of the tasks in the present study was based on previous studies^47,46,54^and adapted to the online data collection platform PsyToolkit^59,60^. Data collection was conducted online due to COVID- 19 pandemic lockdown restrictions. For more details on the procedures, please refer to the Procedure section below.

### Apparatus and stimuli

**Fig. 1:**
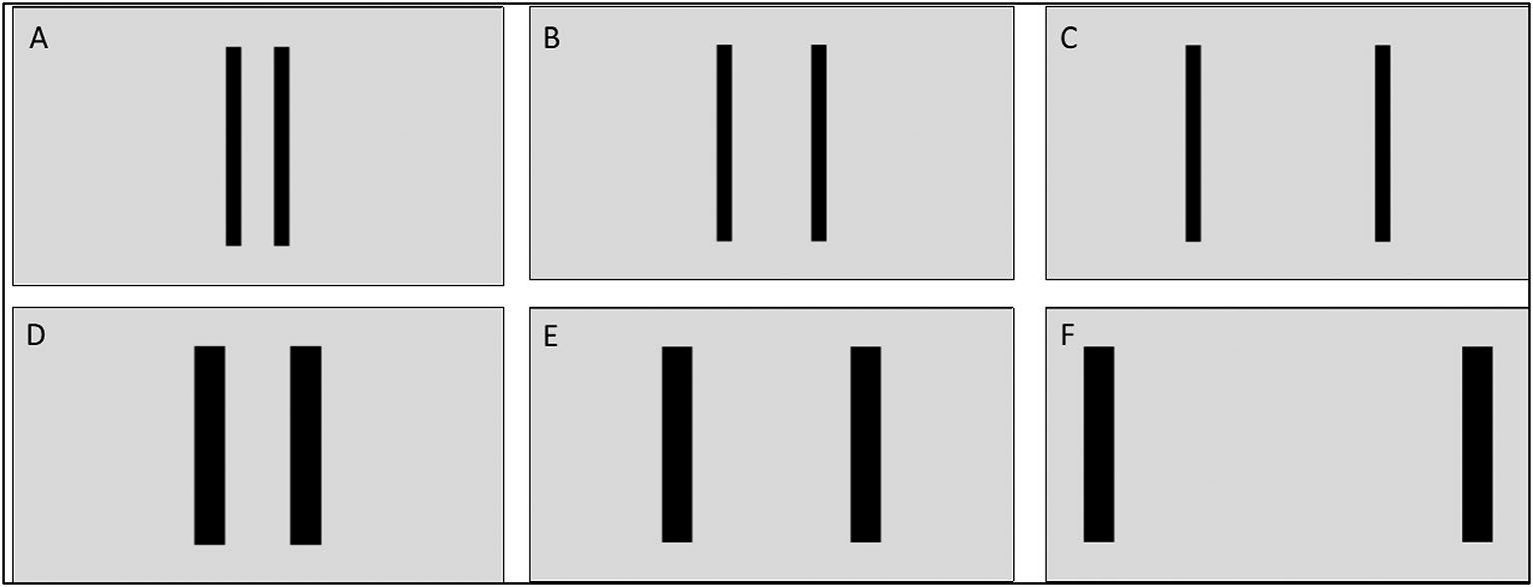
The target pairs. **A**, **B**, **C** are the target pairs with small width and three conditions of distances (short, medium1, and medium2, representing IDs 1, 2, and 3, respectively). **D**, **E**, **F** are the target pairs with large width and three conditions of distances (medium1, medium2, and long; representing IDs 1, 2, and 3, respectively).

Participants completed the experiment on their own computer or laptop in their chosen location, with the researcher communicating via Zoom. Each stimulus was presented on the screen of the computer and consisted of a pair of identical black strips that served as a target pair against a white background. A total of six stimulus images were used for the three tasks. The target pairs in these stimuli varied based on their widths—either small (WSmall) or large (WLarge)—and the distance between them, categorized as short (DShort), medium-short (DMedium1), medium-long (DMedium2), or long (DLong) (see Table 2; Fig. 1). Across all tasks (execution, perception, and imagination), these six combinations of movement distance and target width were utilized to produce three relative index of difficulty (ID) values, namely 1, 2, and 3, as calculated by Fitts’ Law^50^. Each ID had two combinations of target width and movement distance (e.g., ID1 consisted of combinations WSmall-DShort and WLarge-DMedium1). The stimulus images were adaptable to any screen size, and each image was designed with specific “width: distance” ratios: 1:2 for ID1, 1:4 for ID2, and 1:8 for ID3 (see Table 2; Fig. 1). Because of differences in the screen sizes across participants, we focused on consistency in the ratios of target width to movement distance instead of having consistent exact absolute values. Thus, the IDs of 1, 2, and 3 do not refer to the specific calculated ID for each individual, instead they indicate relative increases in ID for each participant’s own individual context. Nonetheless, we asked participants to physically measure and record the movement distances and target widths as well as screen size so we could confirm that the ratios on each screen for each participant were correct and to confirm that the screen size and stimulus characteristics did not differ between groups (Supplementary Table 1, Supplementary Table 2).

All participants began with the action execution task, then proceeded to the perception and imagination tasks. This order was chosen because experience with the execution task has been shown to enhance action perception and imagination^47,46^ and we wanted to provide the conditions in which all participants had the best opportunity to demonstrate intact action imagination. To ensure accurate and consistent performance across all trials and groups in the execution task, clear instructions were provided, and the experimenter monitored participants via video during the task (see Supplementary Table 3).

**Table 2:** Combinations of width and distance to produce the 3 index of difficulty (ID) levels.

IDs		ID 1	ID 2	ID 3
Combination 1	Width	Small	Small	Small
	Distance	Short	Medium1	Medium2
Combination 2	Width	Large	Large	Large
	Distance	Medium1	Medium2	Long
“Width: Distance” ratio		1:2	1:4	1:8

### Tasks

#### Action execution task

**Fig. 2:**
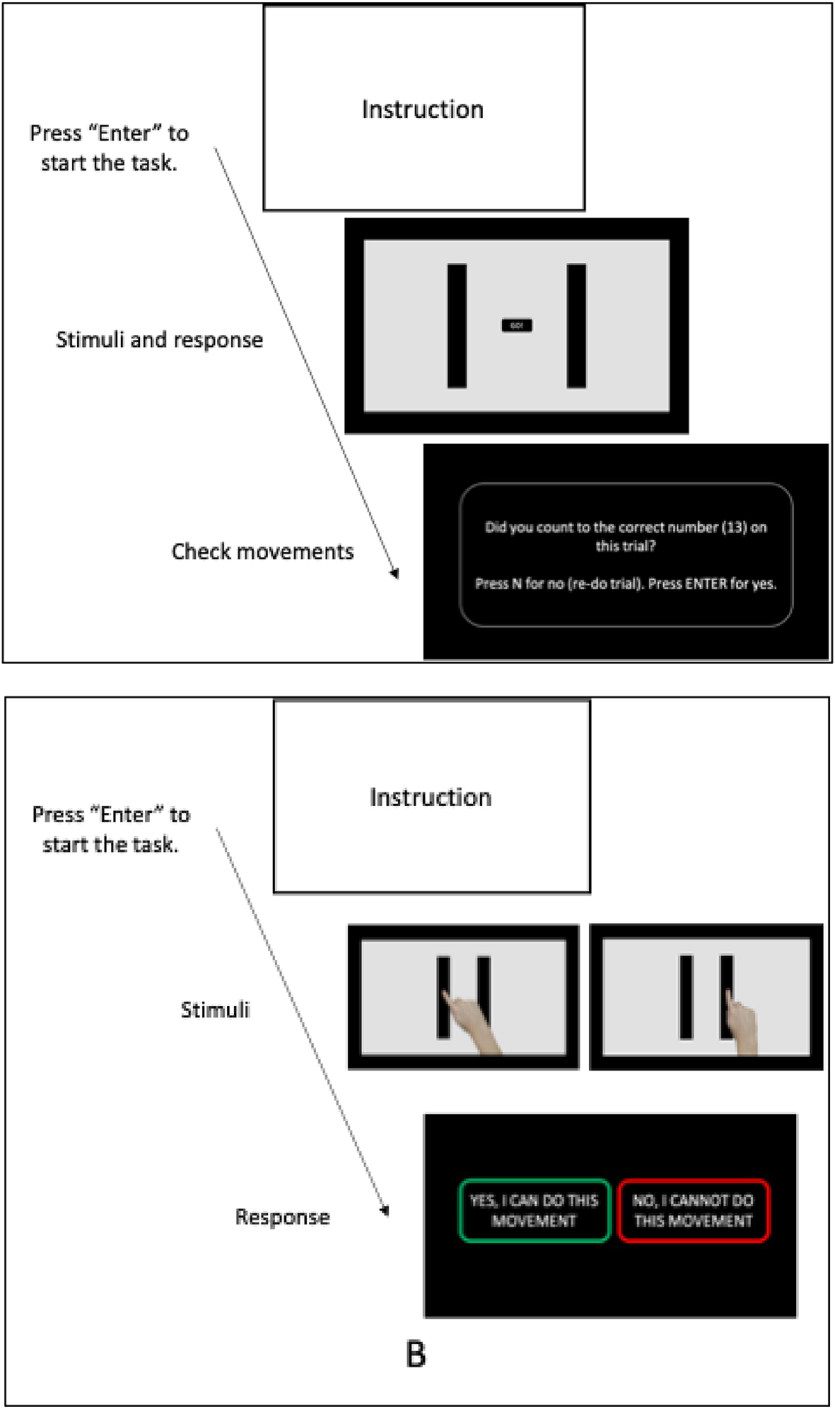
The procedure of the Execution and Imagination task (**A**) and the Perception task (**B**). The hand shown in the Perception task from an adult Caucasian female.

The execution task required participants to perform pointing movements between two target strips. Detailed instructions were displayed (e.g., guidance on what to focus on and how to count their movement times), followed by three practice trials. During this phase, participants had the opportunity to ask questions to the experimenter (who was available via Zoom) to ensure their understanding of the task requirements. The experimenter remained on the video screen throughout testing to ensure task compliance. For each test trial, one of the six combinations of target pairs appeared on the screen, accompanied by a “Ready” sign. Participants were instructed to perform ten rapid and accurate back-and-forth pointing movements between the two target strips, starting from the side corresponding to their dominant hand, using their dominant index finger. Thus, right-handed participants began movements from the right target moving to the left and then back from left to right. Participants were asked to press the spacebar to signal the beginning and end of the ten movements (see Fig. 2A). Specifically, on each trial, participants pressed the spacebar, then moved as quickly and accurately as possible between the right and left target on the screen 10 times, and then moved back to and pressed the spacebar. Accuracy was emphasized, with instructions to land precisely on the middle section of each target while maintaining speed. After completing each movement, participants were asked to confirm whether they had completed exactly ten movements to ensure accuracy in the number of back-and-forth movements performed. This sequence was performed three times per target pair, for a total of 18 trials (6 trials per ID level). Target pairs were presented in a random order.

In addition to the main experimental trials in which reciprocal movements were executed between the 2 targets, participants were also asked to perform 60 control trials (20 trials for each ID) in which they only pressed the spacebar, then touched one target (either on the left or right, depending on participant handedness), and immediately returned to and pressed the spacebar (i.e., without executing 10 movements between the targets). These control trials were completed for each of the target width and location combination to calculate the mean MTs for the reciprocal movements for each condition. Specifically, to compute the average MT for movements between targets alone, the average time to move from the spacebar to the screen and back to the spacebar (indexed via control trials) was first subtracted from the total movement time during the experimental trials (which includes both movements to and from the spacebar to the screen, and the 10 movements between the targets). This approach helped to isolate movement time for moving between the targets, which was then divided by ten to get an average movement time for each individual movement segment between each target. While we were unable to collect detailed spatial movement data during this online task, this method provided an acceptable measure to balance that limitation.

#### Action imagination task

The imagination task was structured similarly to the execution task, but without the physical movement between the targets. In the imagination task, participants were instructed to rest their hand on the table and only position their index finger over the spacebar, without performing any pointing movements. They were asked to imagine themselves making ten accurate pointing movements between the displayed target pairs, as in the previous execution task. To denote the beginning and end of these imagined movements, participants pressed the spacebar once to indicate the start of their imagined movements, and then a second time to indicate the end of their imagined ten movements (see Fig. 2A). After being presented with the instructions, participants undertook three practice trials during which they were asked to count aloud the number of their imagined movements to confirm their understanding. For the actual 18 test trials (6 trials per ID), participants could choose to count the movements either out loud or silently. The duration between the initial and final spacebar presses was recorded. Similar to the execution task, participants also completed 60 control trials, during which they imagined touching one target and pressed the spacebar at the start and end of the imagination task. To compute the average MT for imagining between targets alone, the average time of the control trials was first subtracted from the total imagination time during the experimental trials. This adjusted time was then divided by 10 to calculate the average MT for an individual imagined movement between the targets, using the same method as for the execution task.

#### Action perception task

For the action perception task, participants were asked to watch a series of videos of a hand with an extended index finger (corresponding to their dominant hand) moving back and forth ten times between two targets^46^. Each video was composed of alternating pairs of images of a hand presented with the index finger on the left or on the right target in a first-person view. On a given trial, the images of the hand alternated at a specific time interval to make it appear as though the hand and finger moved with an apparent MT (the time between each image). Although the time interval was consistent within a trial, the time interval differed across trials: 60, 120, 180, 240, 300, 360, 420, 480, 540, or 600 ms. After viewing each video, participants were prompted to judge whether they could replicate the speed and accuracy of the hand in the video: “Can you move at this speed and maintain accuracy?”. They could respond by selecting one of two options shown on the screen following the video: “Yes, I can do this movement” or “No, I cannot do this movement” (see Fig. 2B). Participants completed 120 trials of the perception task (1 trial for each of the apparent MTs presented in both increasing and decreasing orders, for each of the 6 combinations of target width and movement distance; i.e., each combination of a specific time interval for a given combination of movement distance and target width was only observed 2 times). The lowest apparent MT at which participants responded with “Yes, I can do this movement” in the increasing and decreasing sequences was used as the dependent variable; the shortest perceived MT where they judged they could maintain accurate movements^46,51,53^ (similar to the instructions to execute or imagine the shortest MTs while maintaining accuracy). This approach lead to two estimates for each combination of movement amplitude and target width, and 4 per ID, which were then averaged. The proportion of trials where participants selected “Yes, I can do this movement” was 63% for the autistic group and 66% for the non-autistic group. It was necessary to have a larger number of trials for the perception compared to imagination and execution tasks due to the different task structure (i.e., each of the 10 apparent MTs were presented in individual trials across the different combinations of IDs) and need to ensure that we captured a complete range of perceived MTs across multiple sequences.

### Procedure

Participants were asked to position themselves in a quiet room and in front of a computer. They received a Zoom meeting invitation from the experimenter with instructions to enable video for the entirety of the session. All participants agreed and joined the Zoom meeting with screen sharing during the whole experiment session. The primary purpose of screen sharing was for the experimenter to monitor progress and answer questions about the tasks, rather than to review results.

At the start of the Zoom meeting, the experimenter provided an overview of the study and subsequently shared a link for participants to give their electronic informed consent. Additionally, the experimenter checked with participants whether they preferred that the experimenter turned their camera on during the questionnaires and experiment sessions. All participants completed the questionnaire and experiment sessions with the experimenter’s video turned on, allowing the experimenter to observe them throughout the entire session. Once consent was obtained, demographic details such as age, sex, and educational background were collected via Qualtrics (online platform). Then, hand dominance was ascertained using the Edinburgh Handedness Inventory (EHI)^61^. To familiarize participants with the study’s context, a brief video explaining the concept of motor imagery was presented (see : https://osf.io/26azs/?view_only=80cb8ea8c42144e0b95fc03842189583). Following the video, participants had the opportunity to ask the researcher questions, and then they answered questions confirming their comprehension of MI and indicating any prior experience with MI (analysis of the answers to these questions are reported in a separate paper; see^62^). Next, the study utilized an online version of the KVIQ^39^. This online version featured pre-recorded demonstrations of movement tasks, replacing the typical live demonstrations seen in standard in-person KVIQ administration. Participants were guided through the first KVIQ item by the experimenter to ensure that they understood the procedure, then completed the remaining items without assistance (unless requested).

After completing the KVIQ, participants were asked to complete two subscales (Vocabulary and Matrix Reasoning) of the Wechsler Abbreviated Scale of Intelligence (WASI-II)^63^, as well as the SRS-2 and ADC questionnaires. These questionnaires were collected through a Qualtrics survey link. Following the questionnaires, participants were sent another link (via Zoom) to the Fitts’ Law experimental tasks. In these tasks, the participants completed the action execution, perception, and imagination tasks with their dominant hand. At the end of these tasks, participants were instructed to measure and report the exact widths and distances of the targets on their screens, as well as their screen dimensions. Although stimuli were automatically adjusted according to screen size, the measurements were also collected to ensure accurate ID ratio calculations for each individual. The entire session took between 3.5 and 4 hours to complete. Participants had the option to complete the session in a single sitting (*n* = 33, with 17 from the autistic group) or divide it over two consecutive days (*n* = 7, with 3 from the autistic group). A post-hoc test revealed that the mean MT for participants who completed the session in a single sitting was 348 ms, while the mean MT for those who completed it over two consecutive days was 385 ms. There was no significant difference in MT between participants who chose a single session versus two consecutive days (W = 8064.5, *p* = 0.09).

### Analysis

Data were analysed using R (version 4.3.0). The dependent measure was MT for all three tasks – the average length of time it takes for the real (execution task), imagined (imagination task), and observed (perception task) hand to move between target pairs. No outlier procedure was applied to remove data from the perception task. Outliers were detected in the execution and imagination tasks based on the following criteria: (1) If participants report making a mistake during a movement execution or imagination trial, that trial (set of 10 movements) was removed; (2) Data from trials was excluded if the mean MT was < 100 ms (impossibly short MT, and hence likely a recording error) or > 800 ms (atypically long MT and hence the participant is not properly following instructions to achieve fast but accurate movements); and, (3) Outlier removal was performed at the individual and group level and based on the non- recursive procedure recommended by Van Selst and Jolicoeur (1994). Only one execution trial data was removed from a non-autistic participant due to the MT > 800 ms.

Analyses of the three hypotheses presented in the introduction were performed at the group level, and a separate individual-level analysis was carried out as a secondary examination of hypothesis 1. To address hypothesis 1, we sought to determine if the MTs were significantly positively correlated with the three ID conditions for each group for each Fitts’ Law task (action execution, imagination, and perception). Pearson’s correlation coefficients were calculated separately for each task and each group. For hypothesis 2, we aimed to determine if the two groups showed different performance levels for each task. The correlation coefficients were then transformed into *Z* scores, and t-tests were subsequently used to compare these *Z* scores between the two groups for each task. Linear regression analyses were then performed on MTs for each task—execution, imagination, and perception—within each participant group. The components of the regressions between the three IDs and MTs (slope and y-intercept) were compared between the execution, imagination, and perception tasks as in Wong et al.^46^. Y-intercepts were only compared when slopes were not statistically different (for details, see^64^). For hypothesis 3, a mixed ANOVA was conducted to investigate any main effects or interaction effects across Group (autistic and non-autistic group), Task (execution, imagination, perception), and ID (1, 2, 3) in relation to MT. In the mixed ANOVA, Group was treated as a between-subjects variable whereas Task and ID were treated as a within-subjects variable. In an additional analysis, the SRS, ADC, KVIQ-KI and KVIQ-VI were added as covariates to four separate ANOVAs (Task x ID) to test whether these measures affected any potential Group differences in Task performance. If the covariates had an impact on task performance, correlations were calculated to explore these relationships further.

## Results

The normality distribution of variables was tested using Shapiro-Wilk Test (*p* < 0.05). For the autistic group, MT data showed a slight right-skew (skewness = 0.718) and a near-normal kurtosis (− 0.034), but the Shapiro-Wilk test indicated significant non-normality (*p* < 0.001). Similarly, for the non-autistic group, MT data also showed a slight right-skew (skewness = 0.741) and a positive kurtosis (0.163), with the Shapiro-Wilk test confirming significant non-normality (*p* < 0.001). Therefore, non-parametric in addition to parametric analyses were also completed and reported in Supplementary materials.

## Assessment of Fitts’ law in three tasks

To determine if the MTs conformed to Fitts’ Law, Pearson’s correlation coefficients were separately computed for the execution, perception, and imagination tasks for the autistic and non-autistic groups. Consistent with previous studies^46^, MTs were highly and significantly positively correlated with the three ID conditions in all tasks for both groups (see Table 3). Spearman correlation provided similar results (see Supplementary Table 4).

**Table 3:** The relationship between MT (in ms) and ID in all tasks for the autistic and non-autistic groups. MT = movement time, ID = index of difficulty.

Task	Group	
	Autistic group	Non-autistic group
Execution	MT = 295 + 39.5(ID),	MT = 300 + 42(ID),
	*R* = 0.86, *p* < 0.05	*R* = 0.88, *p* < 0.05
Perception	MT = 156 + 55.5(ID),	MT = 181 + 33(ID),
	*R* = 0.94, *p* < 0.05	*R* = 0.90, *p* < 0.05
Imagination	MT = 387 + 28.8(ID),	MT = 334 + 35.9(ID),
	*R* = 0.87, *p* < 0.05	*R* = 0.85, *p* < 0.05

**Fig. 3:**
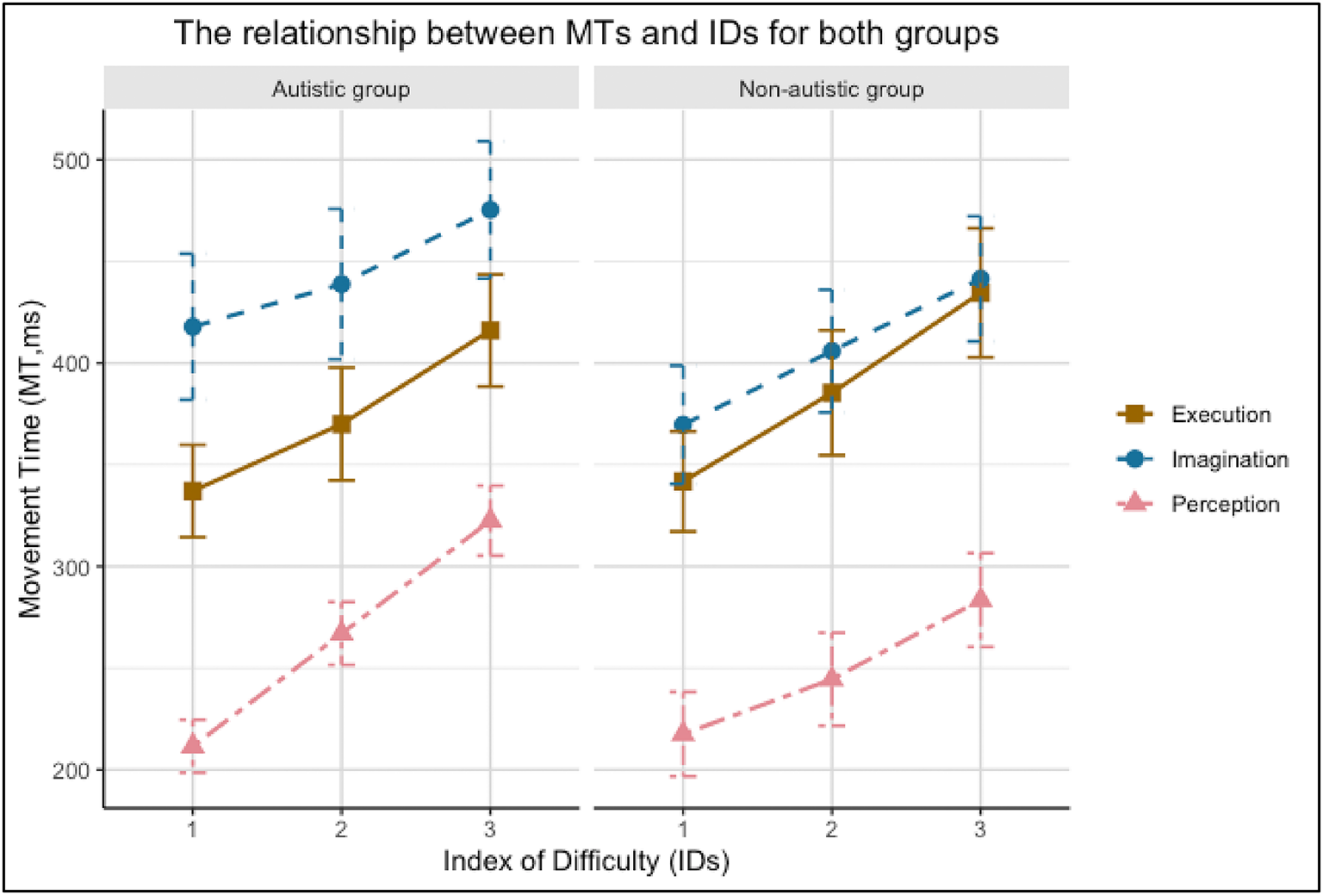
Mean MT as a function of ID for the autistic and non-autistic groups for each task. Fitts’ Law can be intuited from the increase in MT as a function of ID for each task (see Table 3 for details). The lines in the plot represent the progression of mean MTs across increasing levels of ID for each task. Error bars represent standard error of the mean. Brown square with solid line = Execution task, Blue circle with dashed line = Imagination task, Pink triangle with twodashed line = Perception task.

Given the replication of Fitts’ Law across all tasks, linear regression analyses were conducted to compare the correlation coefficients between the autistic and non-autistic groups for each task individually. The correlation coefficient scores were converted to *Z* scores first and then compared between groups for each condition with independent samples *t*-tests. The analyses revealed there was no significant difference in the correlation scores between the two groups in any of the tasks (Execution, *z* = − 0.098, *p* = 0.92; Perception, *z* = 0.29, *p* = 0.78; Imagination, *z* = 0.06, *p* = 0.95). Additional analyses were conducted to compare the components of the regression lines to determine if and how the relationship between MT and ID differed across the tasks, for each group. In these analyses, the components of the regression lines of the group performance on the different tasks were compared to one another based on ratios of the residuals for each line, using separate ANOVAs. The results of these analyses for the group level data revealed that the slopes of the regression lines for the different tasks did not significantly differ in the non-autistic group, *F* (2, 12) = 0.20, *p* = 0.814, nor in the autistic group, *F* (2, 12) = 1.77, *p* = 0.211. The y-intercepts of the lines, however, were statistically different in both the non-autistic group, *F* (2, 14) = 122.84, *p* < 0.001, and the autistic group, *F* (2, 14) = 105.22, *p* < 0.001. Thus, the nature of the differences in the elevations of the lines were elucidated in both groups: MTs were longest in the imagination task, intermediate in the execution task, and shortest in the perception task (see Fig. 3).

## Group comparisons across execution, perception, and imagination tasks

To examine differences between the autistic and non-autistic groups across execution, perception, and imagination, a mixed ANOVA was performed on MTs with a between-subjects factor of Group and within-subjects factors of Task and ID (Greenhouse-Geisser correction were used). There was a significant main effect for Task, *F* (1.77, 67.28) = 29.45, *p* < 0.001, partial *η*^2^ = 0.437; and ID, *F* (1.74, 66.03) = 173.37, *p* < 0.001, partial *η*^2^ = 0.820, but no significant main effect of Group, *F* (1, 38) = 0.30, *p* = 0.589, partial *η*^2^ < 0.001. Post hoc analysis of the main effect of Task (using Tukey’s HSD) revealed that MTs in the imagination task (425 ± 147.8 ms) were longer than those in the execution task (381 ± 127.4 ms), while MTs for the perception task (257 ± 93.4 ms) were shorter than those for the other two tasks. Consistent with Fitts’ Law, the main effect for ID (using Tukey’s HSD) revealed that MTs for ID 1 (316 ± 135.3 ms) were significantly shorter than MTs for ID 2 (352 ± 143.1 ms), which in turn were significantly shorter than MTs for ID 3 (396 ± 141.7 ms).

**Fig. 4:**
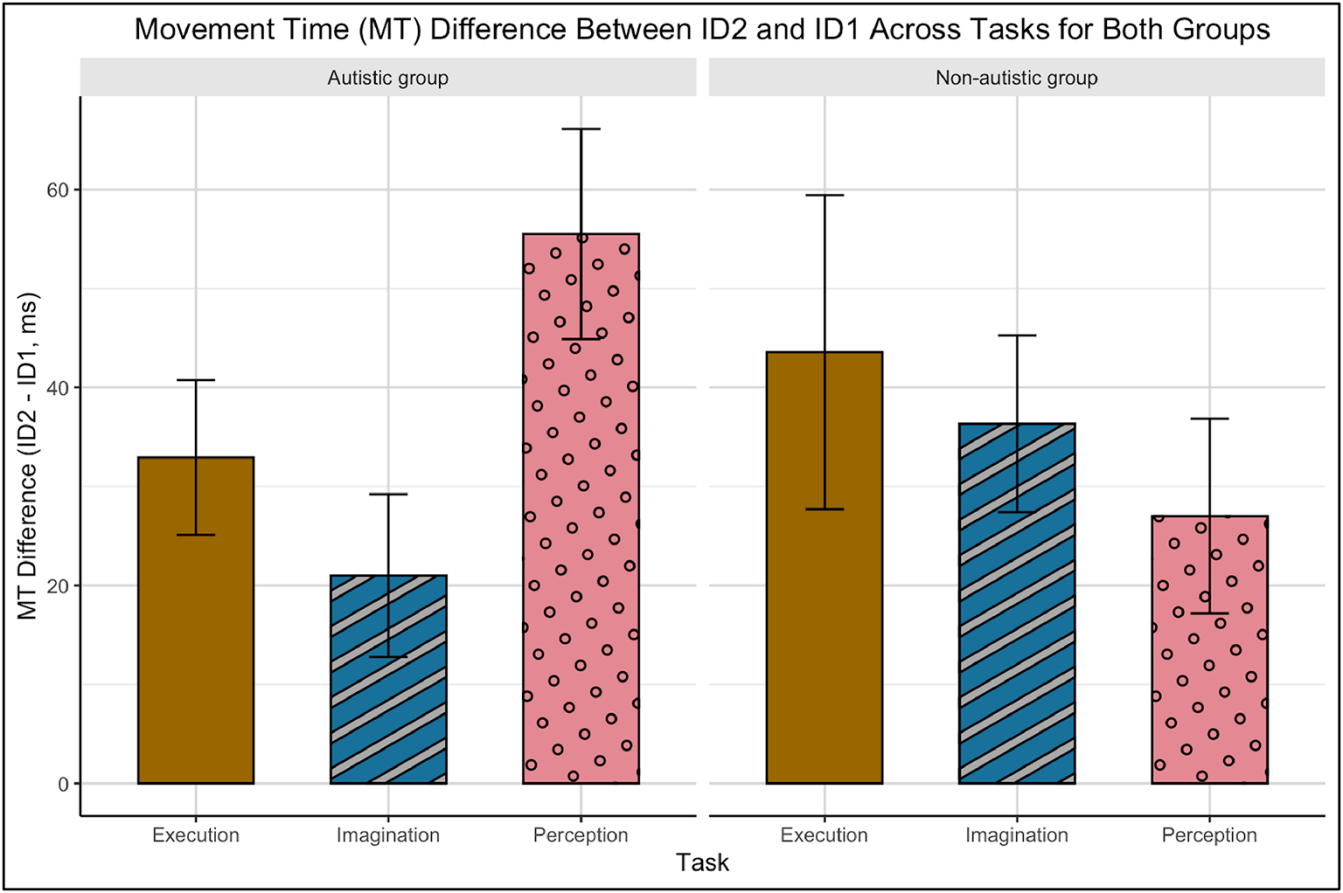
Differences between index of difficulty levels 1 and 2 (ID2 - ID1) across the tasks for the autistic and non-autistic groups. The bar plots display the variability within each task, with the height of each bar representing the mean difference between ID2 and ID1. Black error bars indicate the standard error of the mean (SEM). Brown with solid fill = Execution task, Blue with diagonal stripes = Imagination task, and Pink with polka dots = Perception task.

There were no significant interactions between Group and Task *F* (1.77, 67.28) = 0.65, *p* = 0.505, partial *η*^2^ = 0.017, Group and ID, *F* (1.74, 66.03) = 0.25, *p* = 0.746, partial *η*^2^ = 0.007, or Task and ID, *F* (3.25, 123.41) = 1.55, *p* = 0.201, partial *η*^2^ = 0.039. There was, however, a significant three-way interaction among Group, Task, and ID, *F* (3.25, 123.41) = 2.78, *p* = 0.040, partial *η*^2^ = 0.068. Subsequent post-hoc analysis using two-way repeated measures (Task x ID) ANOVA separately for the two groups revealed a Task x ID interaction for the autistic group only (autistic group: *F* (2.90, 55.23) = 3.84, *p* = 0.015, partial *η*^2^ = 0.006; non-autistic group: *F* (2.76, 52.48) = 0.93, *p* = 0.462, partial *η*^2^ = 0.002) (see Fig. 3 and Supplementary Fig. 1). Specifically, an unexpected, but notable, difference was found between the perception and imagination tasks when the ID increased from 1 to 2 (*p* = 0.023, 95% CI [3.97, 65.04]) – there was a steeper increase in MT from ID 1 to 2 in the perception task than in the imagination task for the autistic group only (see Fig. 4). To further investigate the three way interaction, differences between imagination and execution performance between groups was compared for each ID using t-tests. The results revealed a significant overall group difference in MTs between imagination and execution indicating a larger difference for the autistic group (*t* (115.78) = 2.37, *p* = 0.02). However, when examining group differences for each ID level, the significance diminished (ID1: *p* = 0.29, ID2: *p* = 0.58, ID3: *p* = 0.55, using Bonferroni correction). Mann Whitney and Friedman tests were also conducted and revealed similar results (see Supplementary Fig. 2).

## The covariance effects on Fitts’ law from questionnaire data

A secondary analysis was conducted to assess the impact of scores from the KVIQ, SRS-2, and ADC on performance in tasks adhering to Fitts’ Law. Separate Analysis of Covariance (ANCOVAs) were employed for each set of scores. The results revealed that the inclusion of the KVIQ sub-scores as covariates in the ANOVA did not lead to significant changes in main and interaction effects (see Table 4). However, the earlier observed interaction between Group, Task, and ID was no longer significant after integrating the centred SRS-2 scores and the centred ADC scores as covariates (see Table 4). These analyses suggest that SRS-2 and ADC scores partly explained the interaction effect, indicating that Fitts’ Law performance in the perception versus the imagination task (the source of the 3-way interaction) might be influenced by individuals’ autistic and motor characteristics (see Supplementary Fig. 3 and Supplementary Fig. 4 for more detail).

**Table 4:** ANCOVA results. Changed significant levels indicated in bold.

Questionnaires	ANCOVA results	
KVIQ (VI)	Main effect: Task	*F* (1.77, 65.67) = 28.76, *p* < 0.001
	Main effect: ID	*F* (1.77, 65.67) = 175.00, *p* < 0.001
	Interaction: Group × Task × ID	*F* (3.25, 120.13) = 2.70, *p* = 0.04
KVIQ (KI)	Main effect: Task	*F* (1.75, 64.57) = 29.90, *p* < 0.001
	Main effect: ID	*F* (1.75, 64.57) = 175.00, *p* < 0.001
	Interaction: Group × Task × ID	*F* (3.24, 119.80) = 2.93, *p* = 0.03
SRS- 2	Main effect: Task	*F* (1.76, 25.26) = 28.37, *p* < 0.001
	Main effect: ID	*F* (1.75, 64.43) = 169.44, *p* < 0.001
	**Interaction: Group × Task × ID**	***F*** **(3.19**,** 118.02) = 2.03**, ***p*** **= 0.11**
ADC	Main effect: Task	*F* (1.73, 64.16) = 29.25, *p* < 0.001
	Main effect: ID	*F* (1.75, 64.63) = 169.05, *p* < 0.001
	**Interaction: Group × Task × ID**	***F*** **(3.23**,** 119.46) = 0.95**, ***p*** **= 0.43**

KVIQ: Kinesthetic and Visual Imagery Questionnaire (VI: Visual Imagery, KI: Kinesthetic Imagery), SRS-2: Social Responsiveness Scale, Second Edition, ADC: Adult Developmental Coordination Disorders/Dyspraxia Checklist.

## Individual analysis of execution, perception, and imagination

Consistent with the Group level analysis of the slopes and y-intercepts of the regression lines, we tested whether the group level pattern of effects was also present on the individual level. Results revealed that, although there was some variation between individuals, the overarching trend was that the processes underlying action execution, perception, and imagination are largely consistent among participants, regardless of their group status. Full details are reported in the Supplementary materials (see Supplementary Table 5, Supplementary Table 6, Supplementary Table 7, Supplementary Fig. 5 and Supplementary Fig. 6).

## Discussion

The goal of this work was to investigate whether autistic adults can engage in explicit MI, and how MI processes relate to action execution and perception in both autistic and non-autistic individuals. This study builds on previous research^46^ involving non-autistic individuals by comparing executed, perceived, and imagined MTs within a Fitts’ Law paradigm. Consistent with Wong et al.^46^, our study demonstrated a similar Fitts’ Law relationship across all three tasks for both autistic and non-autistic groups, with MTs increasing as the ID levels increased. Despite some variability among participants, similar performance patterns emerged at the individual level across both groups. These findings align with the common coding hypothesis, which posits that action execution, perception, and imagination are related through shared, abstract neural codes that facilitate a bidirectional response, enabling individuals to interact with their environment effectively^13,15^. In sum, our results suggest that MI is relatively intact in autistic individuals, as shown by the similarities in MTs between autistic and non-autistic groups during the imagination task.

First and foremost, Fitts’ Law was observed across all tasks in both groups of participants. The imagination task yielded the longest MTs for both groups^46 ^(although the difference between this and the execution task was larger for the autistic group, see below). This longer MT for imagined movements may be attributed to the additional cognitive load involved in maintaining imagined movements, such as working memory demands^20,65^, as proposed by Glover et al.^57^. Supporting this interpretation, research has shown that the dorsolateral prefrontal cortex, associated with movement inhibition, is activated during motor imagination but not during action execution or perception, reflecting the increased effort to suppress actual movement during imagination^20,66^.

It should be noted that a significant three-way interaction among Group, Task, and ID was also observed, driven by Task and ID effects within the autistic group, revealing a potentially spurious difference between perception and imagination tasks. This result is somewhat incongruent with the results of the regression analysis in which no differences in the slopes of the regression lines were detected and should be interpreted with caution due to the non-normality of the data. Nonetheless, post hoc analysis revealed that the autistic group displayed a more pronounced increase in MT between ID 1 and 2 for perception relative to imagination. Moreover, the autistic group showed a greater increase in MT during imagination compared to execution than the non-autistic group, although further analysis found no significant group differences at any specific ID level. This discrepancy indicates that, in comparison to referencing actual movements, the autistic group may utilize alternative strategies or cognitive processes for completing the imagination task, echoing the suggestions of previous studies^9,42,67,68^. Caution is warranted, however, when interpreting these results due to the potential influence of group characteristics, when SRS-2 and ADC scores were included as covariates, the interaction effects in Fitts’ task performance disappeared, suggesting that social ability and motor characteristics might influence the differences in performance in perception and imagination tasks.

The overarching findings of similarities in performance of MT and IDs for the perception and execution tasks between the autistic and non-autistic group is consistent with literature indicating that autistic individuals have comparable abilities in movement speed perception tasks^22,69,70^ and Fitts’ Law relationship for simple hand aiming movements^56^. The current study utilized mental chronometry to assess explicit MI, and despite lower ADC scores, individuals with autism exhibited proficiency in the imagination task. Previous research exploring explicit MI in autism, using self-reported questionnaires or experimental tasks, has yielded mixed results^9,41–43^. One study using a bimanual drawing task, found that unlike the non-autistic individuals, autistic individuals were unaffected by imagining drawing a different shape with their other hand^42^. Another study reported that autistic individuals did not benefit from imagining a sequence in a sequence recall task^46^. However, the current study observed a similar Fitts’ Law relationship in both groups for the imagination task. The disparity could be attributed to the simplicity of our task design, requiring participants to focus solely on the MI, without engaging in other cognitive processes. Alternatively, a limitation of the previous studies^42,43^ is that it is unclear whether autistic individuals were actually using MI as they potentially did not need to use it to complete the tasks. In contrast, MI is more central and “less optional” in chronometry tasks, as participants are asked to report the duration of the imagined movement.

Moreover, in contrast to the Fitts’ tasks performance, significant group differences were found in both the VI and KI subscales of the KVIQ, with the autistic group reporting less vivid imagery. This result contrasts with a previous study reporting no significant differences between autistic and non-autistic groups^41^. One potential explanation for this discrepancy may be that the current study utilized a video-recorded short version (5-item) of the KVIQ, whereas Gowen et al.^41 ^employed an in-person full version. In the in-person setting, having participants sit side by side with the experimenter may have been more conducive to facilitating MI and have a full 3D rendering of the movements, as opposed to third person demonstrations of movements in the online version. Some participants in the current study reported that they could only imagine the actor in the video performing the movements, rather than visualizing themselves executing the tasks^62^. This difference in perspective could significantly impact how participants engage with and perform in MI tasks.

Comparing the imagination results from the Fitts’ task and KVIQ questionnaire, the different results may also be due to the differing methodologies assessing different aspects of MI, with the self-reported KVIQ focusing more on how participants generate motor images, and the Fitts’ task emphasizing the capability to maintain motor images^71^. Meanwhile, participants may utilize alternative strategies during the imagination task, such as moving their eyes between targets (such eye movements were anecdotally observed by the researcher, but not recorded during the experiment^72^). Further studies could explore if autistic individuals can demonstrate MI when asked to not move their eyes (note that restricting eye movements impacted imagined MTs in non-autistic individuals^72^). Additionally, autistic individuals might express their internal experiences less proficiently or exhibit increased caution when responding to subjective scales^73,74^. A further reason for the divergent findings may be the different movements involved. While the KVIQ includes a variety of movements, such as lifting an arm, bending the upper body, or lifting a leg, the imagination task involved only aiming with the dominant hand. These could suggest that while autistic individuals may be adept at simple movements, they may struggle with more complex tasks and potentially employ different strategies compared to non-autistic individuals^4,62^. Finally, it is important to acknowledge individual differences in MI; when KVIQ scores were considered as a covariate, they did not significantly alter performance on Fitts’ task.

Our study’s findings suggest that individuals with autism are capable of effectively utilizing MI, indicating potential for further exploration of MI as a therapeutic approach. While more research is needed, particularly in areas such as kinematics and real-world application, the use of MI in conditions like Developmental Coordination Disorder^75^, Parkinson’s disease^76,77^, and post stroke^80 ^rehabilitation suggests it could be a promising tool to support daily life task performance in autistic individuals. It is important to note that all participants completed the execution task first, providing recent sensory and proprioceptive input, potentially enhancing their performance in subsequent tasks (MI and perception). From an applied perspective, we recommend incorporating action observation and physical movement (if possible) before engaging in MI, as seen in other interventions such as action observation motor imagery^75,76,79,80^. This approach may be particularly beneficial for autistic individuals, who may rely more on proprioceptive input^81–83^.

Our study successfully replicated the findings of Fitts’ tasks in non-autistic groups in an online environment. However, conducting the experiment in an online setting introduced certain limitations, and caution is needed when interpreting our results. The lack of spatial and kinematic data collection of the actual hand movements during execution meant that our analysis was confined to movement time. The absence of movement accuracy results in a potential limitation only for the execution task because Fitts’ Law is typically applied when movements accurately terminate on the targets 95% of time. Although the presence of the Fitts’ Law relationship between MT and ID in the execution task suggests that this level of accuracy was achieved, we were not able to confirm this movement accuracy. Note, however, that this limitation in recording accuracy only impacts the movement execution task because movements were 100% accurate in the video used in the perception task and accuracy can never be assessed in the imagination task (due to the absence of overt movement). The absence of the kinematic data in the imagination task also prevented us from exploring additional non-goal small hand movements (motor overflow) during the imagination task, which have previously been found to increase as the amplitude of the imagined movements increased^46,72^. Although not central to testing the main research hypothesis, this analysis might have provided deeper insight into motor system activation during motor imagery in our groups. Additionally, as this is the first study to explore motor cognitive processes in autism, our initial power analysis was based on a simpler model, which may not fully account for the added complexity of the final analyses and may restrict the study’s ability to detect more subtle interactions or small effects. The online design also required participants to have internet access and a certain level of comfort with computers and one-on-one interactions with the experimenter, meaning the autistic individuals who chose to volunteer were relatively young and verbal. Future research conducted in a controlled laboratory setting would enable more comprehensive data collection, including metrics such as movement accuracy, smoothness, and motor overflow. Moreover, introducing more complex tasks and using power analyses aligned with the complexity of the intended models could provide deeper insights into whether the observed performance equivalence between autistic and non-autistic individuals persists under more demanding conditions.

Due to the constraints of our study’s settings and procedures, we were unable to thoroughly investigate the specific strategies employed by individuals during the tasks. The exploration of these personal MI strategies and motor characteristic and their impact on task performance remains a significant area for future research. It is particularly important to investigate the alternative strategies that autistic individuals might use during MI tasks (e.g., counting, moving eyes, small finger movements, etc.)^62^. Understanding these strategies could be instrumental in developing MI-based interventions tailored to improve motor functions in individuals with autism, potentially leading to innovative therapeutic approaches that leverage the unique cognitive profiles of this population.

In conclusion, the current study has demonstrated that individuals with autism exhibit Fitts’ Law across perception, execution and imagination tasks and effectively engage in explicit MI. Further research should delve deeper into explicit MI strategies used in autism, particularly in more controlled experimental settings, to validate these findings and to inform potential imagery-based interventions.

## Electronic supplementary material

Below is the link to the electronic supplementary material.

Supplementary Material 1

## Data Availability

Once published, the corresponding author, E.G., will make the data publicly available on Open Science Framework in a way that safeguards participants’ privacy and ensures that no identifying information can be traced back to individual participants.
