# Supplementary material for: Speed-accuracy trade-offs in action perception, motor imagery, and execution of hand movements in autistic and non-autistic adults
Source: Sci Rep. 2025 Apr 17;15:13255. doi: 10.1038/s41598-025-97036-w (PMC12006536; doi:10.1038/s41598-025-97036-w)
Supplement: Supplementary file 1 — Supplementary Material 1 [file 41598_2025_97036_MOESM1_ESM.docx]

**Supplementary Materials**

## **Sensitivity analysis**

## We conducted a sensitivity analysis used G*Power to conduct post-hoc power calculations based on the observed effect sizes from our mixed ANOVA model. The analysis was based on a sample size of 20 participants for each group (autistic and non-autistic) and 3 tasks. For the main effect of Group, the observed effect size was f = 0.088, resulting in a power estimate of 0.10. For the main effect of Task, the observed effect size was f = 0.886, leading to a power estimate of 1.00. Lastly, for the Group × Task interaction, the observed effect size was f = 0.143, resulting in a power estimate of 0.48. These results indicate that our study is well-powered to detect the main effect of Task, but underpowered to detect the main effect of Group and the Group × Task interaction.

## **Variability in screen size and target conditions**

1. Screen size

The screen size was 15.61 ± 3.75 inches (range: 13 to 24 inches) in the autistic group and 14.39 ± 3.22 inches (range: 11 to 24 inches) in the non-autistic group.

1. Movement Distances and Target Widths

There were instances where data on movement distances and target widths were missing due to internet and online experiment application errors. Specifically, 2.5% of the width data (from 1 non-autistic participant) and 7.5% of the distance data (from 1 autistic and 5 non-autistic participants) were affected. Despite these missing data, the actual measurements of movement distance and target width were consistent with our condition settings (see Table 1). The width-to-distance ratios for each ID were as follows: ID1 = 1:2, ID2 = 1:4, and ID3 = 1:8.

Table 1.Mean and standard deviation (Mean ± SD) of target width and movement distance across different ID levels and conditions.

| IDs  (width: distance) | Conditions | Width (cm)  Mean ± SD | Distance (cm)  Mean ± SD |
| --- | --- | --- | --- |
| ID 1  (1:2) | Condition 1 | 0.93 ± 0.33 | 2.86 ± 0.57 |
|  | Condition 2 | 1.78 ± 0.43 | 5.73 ± 0.99 |
| ID 2  (1:4) | Condition 3 | 0.90 ± 0.32 | 5.72 ± 0.83 |
|  | Condition 4 | 1.81 ± 0.41 | 11.19 ± 0.88 |
| ID 3  (1:8) | Condition 5 | 0.93 ± 0.37 | 11.26 ± 1.39 |
|  | Condition 6 | 1.78 ± 0.41 | 22.62 ± 2.78 |

1. Comparison of group difference in screen size, target distance and width

We conducted t-tests to test whether screen size, target distance, and width differed significantly between the autistic and non-autistic groups. The results indicated no significant differences for any of the conditions. Details in the Table 2.

Table 2. Mean, standard deviation (Mean ± SD) and group comparisons of screen size, target width, and distance.

| Variables | Autistic group (Mean ± SD) | Non-autistic group (Mean ± SD) | t-statistic | p-value |
| --- | --- | --- | --- | --- |
| Screen Size | 15.61 ± 3.75 | 14.39 ± 3.22 | 1.10 | 0.278 |
| Distance Short | 2.87 ± 0.46 | 2.85 ± 0.68 | 0.08 | 0.938 |
| Distance Medium 1 | 5.75 ± 0.77 | 5.70 ± 1.05 | 0.17 | 0.862 |
| Distance Medium 2 | 11.27 ± 1.57 | 11.17 ± 1.66 | 0.19 | 0.850 |
| Distance Long | 23.01 ± 2.50 | 22.18 ± 3.09 | 0.88 | 0.385 |
| Width Small | 0.90 ± 0.12 | 0.94 ± 0.46 | -0.39 | 0.698 |
| Width Large | 1.82 ± 0.25 | 1.76 ± 0.53 | 0.41 | 0.683 |

## **Variability in Measurements**

For all three tasks (Execution, Perception, and Imagination), the results of Levene's test show that there is no significant difference in the variability (standard deviation) of MT between the autistic and non-autistic groups (see Table 3). This suggests that both groups exhibit similar levels of variability in their responses across the tasks.

Table 3. Mean, standard deviation (mean ± SD) and variability comparison of MT across Execution, Perception, and Imagination tasks between both groups. MT reported in ms.

| Task | Group | | Levene's test |  |
| --- | --- | --- | --- | --- |
|  | Autistic group  mean ± SD | Non-autistic group  mean ± SD |  |  |
| Execution | 467.43 ± 41.26 | 485.07 ± 49.02 | *F* (1, 118) = 0.481, *p* = 0.4895 |  |
| Perception | 267 ± 52.92 | 247 ± 32.69 | *F* (1, 118) = 1.677, *p* = 0.1979 |  |
| Imagination | 608.83 ± 33.70 | 538.92 ± 41.18 | *F* (1, 118) = 2.200, *p* = 0.1407 |  |

## **Non-parametric results**

1. Assessment of Fitts’ Law in three tasks

Spearman’s correlation coefficients also separately computed for the execution, perception, and imagination tasks for the autistic and non-autistic groups and revealed same results as Person’s correlation (see Table 4).

Table 4. The Spearman correlation between MT (in ms) and ID in all tasks for the autistic and non-autistic groups. MT = movement time, ID = Index of Difficulty.

| Task | Group | |
| --- | --- | --- |
|  | Autistic group | Non-autistic group |
| Execution | MT = 295 + 39.5(ID), | MT = 300 + 42(ID), |
|  | *R* = 0.96*, p* < 0.01 | *R* = 0.96*, p* < 0.01 |
| Perception | MT = 156 + 55.5(ID), | MT = 181 + 33(ID), |
|  | *R* = 0.96, *p* < 0.01 | *R* = 0.96, *p* < 0.01 |
| Imagination | MT = 386 + 28.8(ID), | MT = 334 + 35.9(ID), |
|  | *R* = 0.84, *p* < 0.05 | *R* = 0.96, *p* < 0.01 |

1. Group comparisons across execution, perception, and imagination tasks

Same as the ANOVA results, in comparing the main effects of Group, the Mann-Whitney test revealed no significant group differences (*W* = 17143, *p* = 0.34).

Friedman test was conducted to assess differences in MT across the Task (Execution, Imagination, and Perception) and IDs (1, 2, and 3) for both autistic and non-autistic groups separately (Fig.1). For the autistic group, the Friedman test revealed a significant difference in MT across tasks, χ² (2) = 19.9, *p* < 0.001. Post-hoc pairwise comparisons were conducted using the Wilcoxon signed-rank test with Bonferroni correction applied to adjust for multiple comparisons between tasks showed a statistically significant difference between Execution and Imagination tasks *V* = 35, *p* = 0.022 (adjusted), Execution and Perception tasks, *V* = 192, *p* = 0.001 (adjusted), and Imagination and Perception tasks, *V* = 202, *p* < 0.001 (adjusted). Friedman test also revealed a significant difference in MT across IDs in autistic group, χ² (2) = 40, *p* < 0.001. Post-hoc pairwise comparisons were conducted using the Wilcoxon signed-rank test with Bonferroni correction applied to adjust for multiple comparisons between IDs showed a statistically significant difference between ID 1 and ID 2, *V* = 0, *p* < 0.001 (adjusted), ID 1 and ID 3, *V* = 0, *p* < 0.001 (adjusted), and ID 2 and ID 3, *V* = 0, *p* < 0.001 (adjusted).

For the non-autistic group, the Friedman test revealed a significant difference in MT across tasks, χ² (2) = 13.9, *p* < 0.001. Post-hoc pairwise comparisons were conducted using the Wilcoxon signed-rank test with Bonferroni correction applied to adjust for multiple comparisons showed a statistically significant difference between Execution and Perception tasks, *V* = 184, *p* = 0.006 (adjusted), Imagination and Perception tasks, *V* = 198, *p* < 0.001 (adjusted), but no difference between Execution and Imagination tasks *V* = 80, *p* = 1.000 (adjusted). Friedman test also revealed a significant difference in MT across IDs in the non-autistic group, χ² (2) = 34.3, *p* < 0.001. Post-hoc pairwise comparisons were conducted using the Wilcoxon signed-rank test with Bonferroni correction applied to adjust for multiple comparisons between IDs showed a statistically significant difference between ID 1 and ID 2, *V* = 5, *p* < 0.001 (adjusted), ID 1 and ID 3, *V* = 0, *p* < 0.001 (adjusted), and ID 2 and ID 3, *V* = 5, *p* < 0.001 (adjusted).


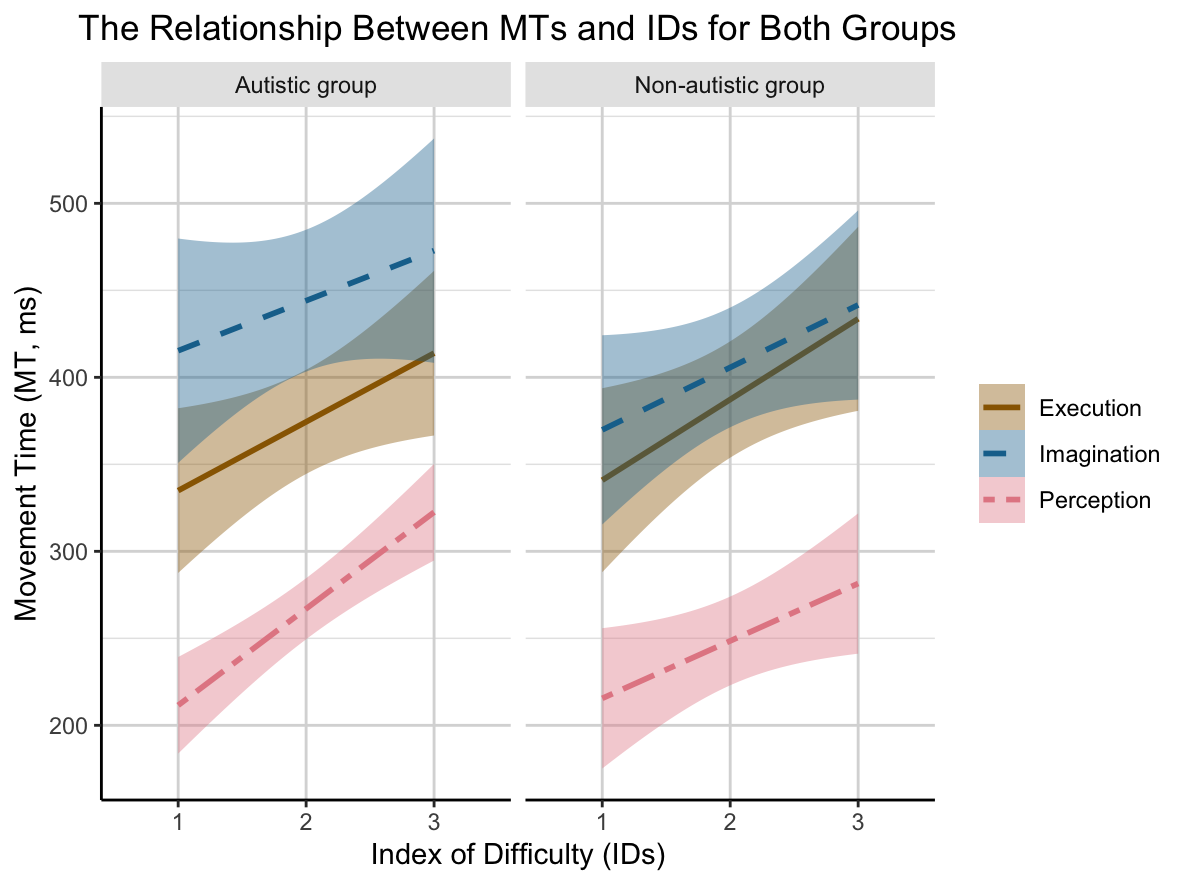


Fig 1.The relationship between MTs and IDs for autistic and non-autistic groups. The plot shows regression lines for three tasks, with shaded areas representing 95% confidence intervals around each line. Separate panels illustrate the autistic group (left) and the non-autistic group (right). The lines represent the fitted regression slopes for each task within each group. Brown solid line and shaded area = Execution task, Blue dashed line and shaded area = Imagination task, Pink twodashed line and shaded area = Perception task. The y-axis indicates MT (in milliseconds), and the x-axis denotes ID levels (1, 2, 3).

1. Group comparisons between execution and imagination tasks

Same as t-test results, in comparing the group differences in MTs (imagination - execution) under each ID level, the Mann-Whitney U test revealed the overall group difference (*W* = 2209, *p* = 0.03), but no significant group differences were observed at any specific ID level (ID1: *p* = 0.29, ID2: *p* = 1.000, ID3: *p* = 0.87, using Bonferroni correction). The overall group difference may result from the cumulative small differences across all ID levels. However, when analysed separately, the small differences within each ID level may not have reached significance. This indicates that the observed group difference is distributed across IDs rather than being specific to any single ID level (Fig 2).


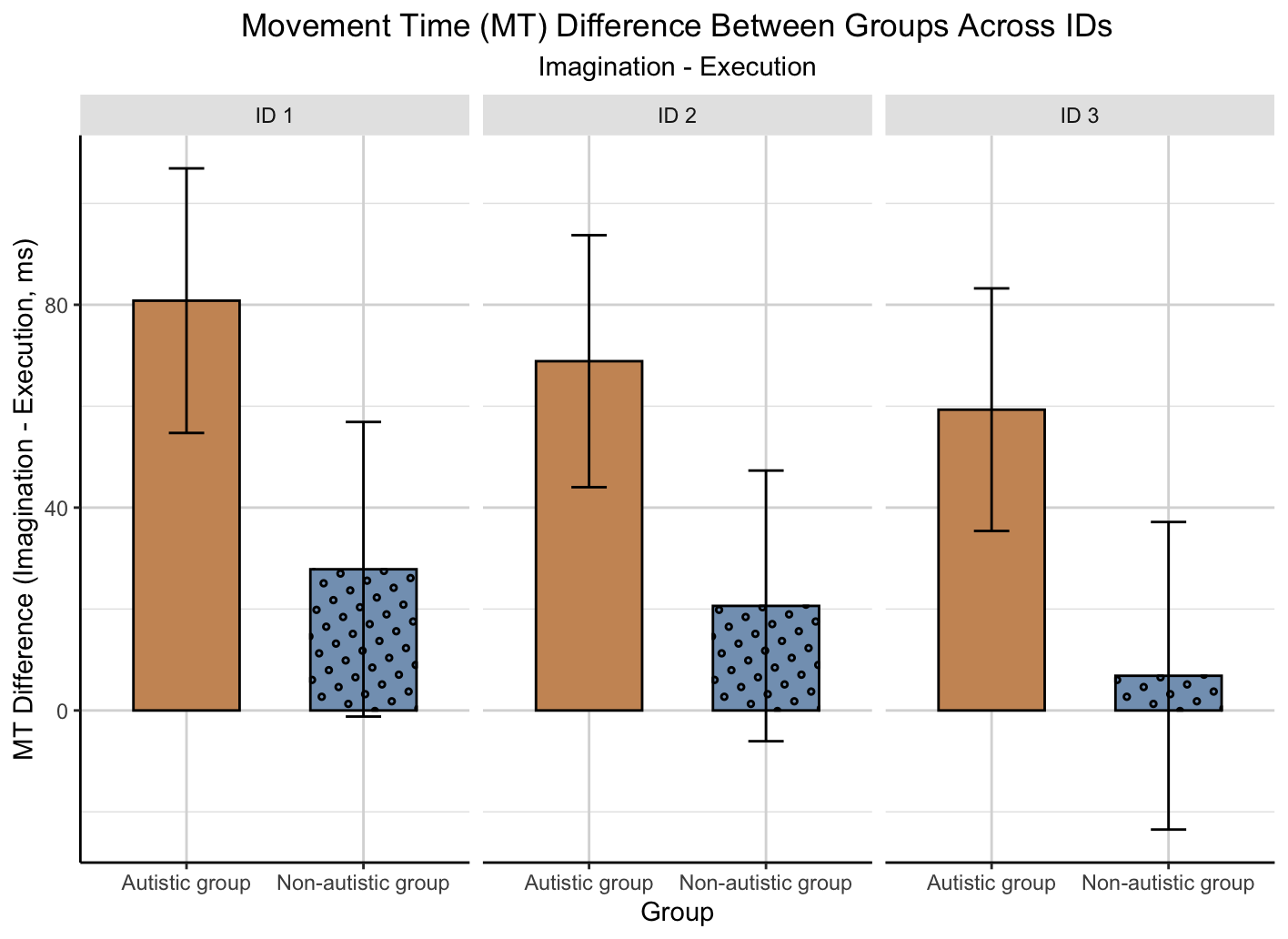


Fig 2. Movement Time (MT) Difference Between Groups Across IDs in Imagination and Execution Tasks. The bar plot shows the difference in MT between imagination and execution tasks across IDs for the autistic and non-autistic groups. The y-axis represents the MT difference (in milliseconds), while the x-axis indicates group differences for each ID level. Error bars indicate the standard error of the mean (SEM). Brown solid fill bars = Autistic group, and blue polka dot bars = Non-autistic group.

## **The effect of SRS-2 and ADC scores on the Movement Time**

1. Effects of SRS-2 on Movement Time


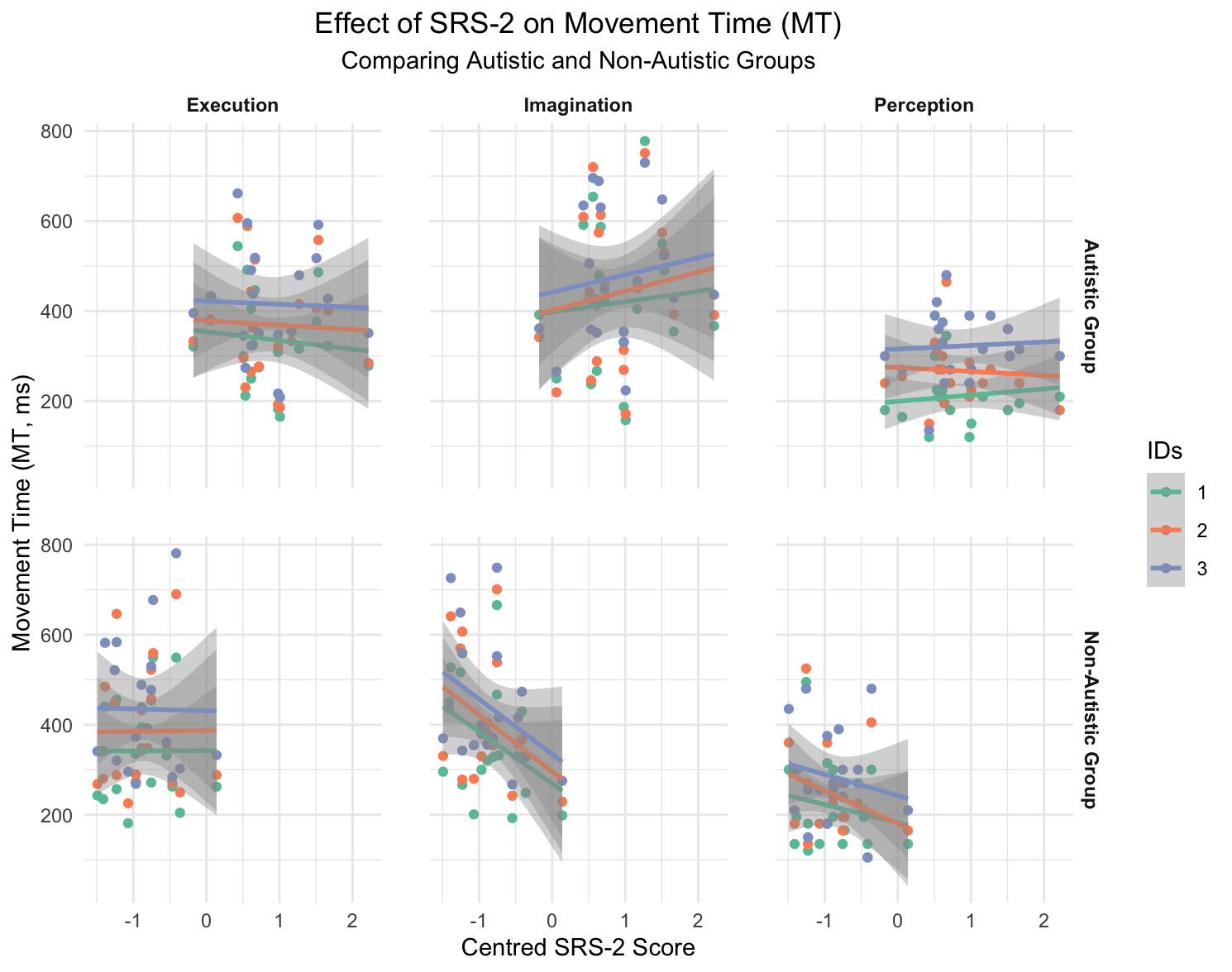


Fig 3. The plot shows the effect of SRS-2 on movement time (MT) across three tasks: Execution, Imagination, and Perception. Each panel represents a different combination of task and group (Autistic and Non-Autistic groups). Lines and points are color-coded by ID level: green for ID 1, orange for ID 2, and blue for ID 3. The y-axis shows Movement Time (MT) in milliseconds, while the x-axis displays the centred SRS-2 score. The shaded areas around the lines indicate 95% confidence intervals for the regression line in each task and group combination.

1. Effects of ADC on Movement Time


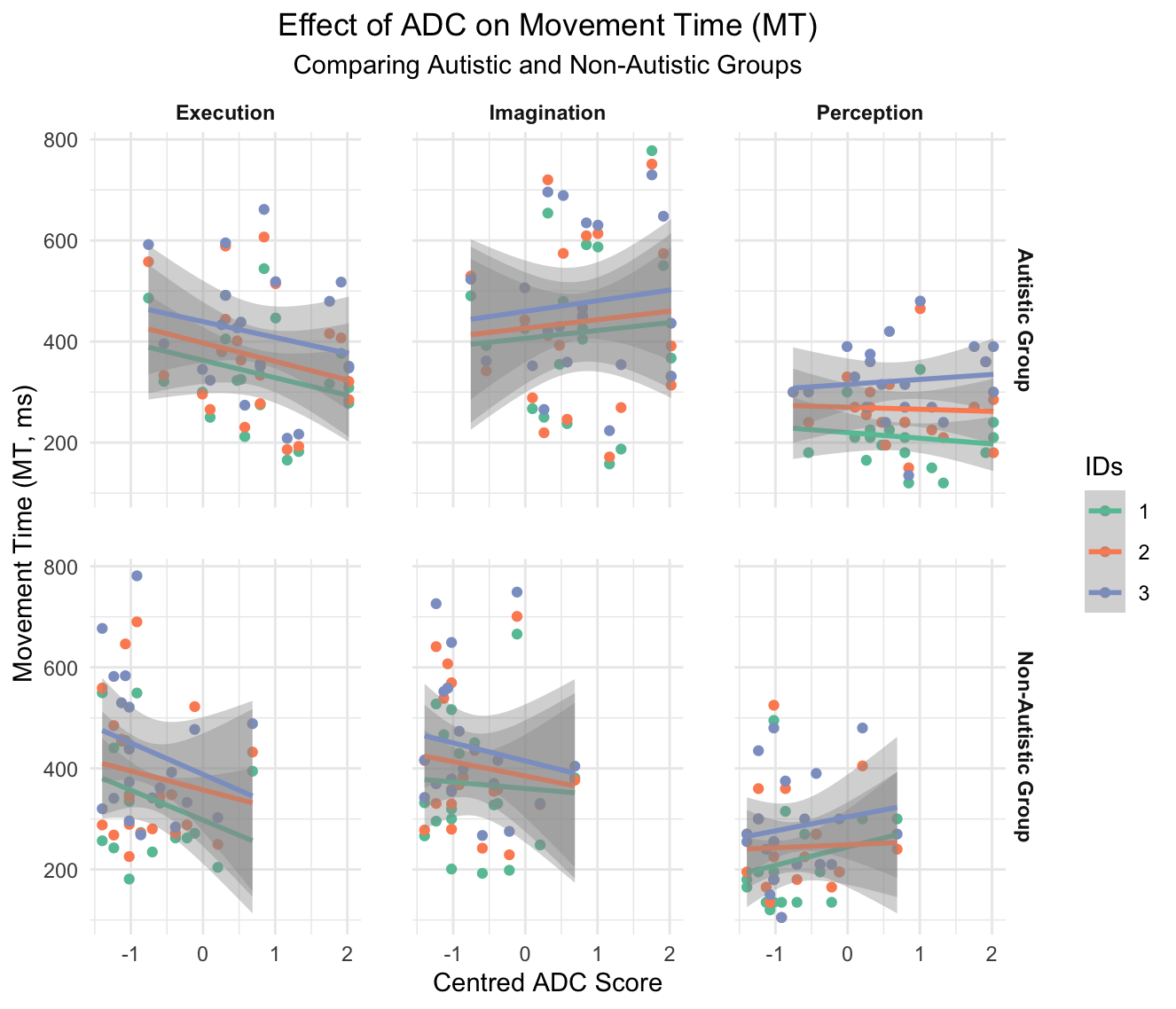


Fig 4.The plot shows the Effect of ADC on Movement Time (MT) across Execution, Imagination, and Perception tasks. Each panel represents a different combination of task and group (Autistic and Non-Autistic groups). Lines and points are color-coded by ID level: green for ID 1, orange for ID 2, and blue for ID 3. The y-axis shows Movement Time (MT) in milliseconds, while the x-axis displays the centred ADC score. The shaded areas around the lines indicate 95% confidence intervals for the regression line in each task and group combination.

## **Individual level comparisons across execution, perception, and imagination**

The results of the group-level data generally show that the processes underlying action execution, perception, and imagination are intact in the autistic group. A more rigorous test of the hypothesis that the perception-action representations underlying all three abilities are intact in autistic people would be to assess whether the group-level pattern of effects is also present on the individual level. To this end, a series of additional analyses were conducted in which the slope and y-intercept components of the linear regressions between MT and ID for each individual participant in each task were compared (see also^1^). For the sake of brevity and clarity, none of the regression equations and associated statistics will be reported here. Instead, only summary data regarding the number and direction of significant differences between the slopes and y-intercepts of the regression lines for the three tasks will be reported. For these analyses, alpha was adjusted to 0.01 in consideration of the multiple sets of comparisons for each participant that were conducted. Note that y-intercepts were only compared when the slopes were not different because one cannot reliably compare y-intercepts when the slopes are different. If slopes are statistically different, this implies a significant difference in the relationship between the variables being measured. In such cases, comparing y-intercepts would not be meaningful, as the interaction between variables alters the overall relationship. If slopes are not significantly different, this suggests similar performance scaling between groups or conditions. In this scenario, comparing y-intercepts helps determine whether there are baseline differences between groups. Figures 1 and 2 graphically illustrate the data for each individual participant in the autistic and non-autistic group in the end.

1. Execution and Imagination

The first set of analyses involved comparisons between the data for the MTs in the execution and imagination tasks. Consistent with the main analysis, the comparisons between the individual regression lines revealed that the regressions for the majority of the participants had slopes that did not differ, but higher y-intercepts on the imagination task than on the execution task. Specifically, the comparisons of the regression lines between the imagination and execution MTs revealed that the slopes for the participants were not significantly different for 18 of the 20 participants in the autistic group and for all 20 of the 20 participants in the non-autistic group. Of the 18 autistic participants whose slopes did not differ, 8 had higher y-intercepts for imagination MTs than execution MTs, 2 had a lower y-intercept for imagination MTs than execution MTs, and 8 had y-intercepts which did not differ between imagination MTs and execution MTs. For the 20 participants in the non-autistic group whose slopes did not differ, 9 had higher y-intercepts for imagination MTs than execution MTs, 4 had lower y-intercepts for imagination MTs than execution MTs, and 7 had y-intercepts which did not differ between imagination MTs and execution MTs (see Table 5).

Table 5. Slope and y-intercept differences between execution and imagination tasks

| Autistic group | Execution-Imagination | | Non-autistic group | Execution-Imagination | |
| --- | --- | --- | --- | --- | --- |
|  | Slope  (ms/ID) | y-intercept  (ms) |  | Slope  (ms/ID) | y-intercept  (ms) |
| ASC_01 | 0.79 | -29.45^*^ | N-ASC_01 | 0.84 | -404.09^*^ |
| ASC_02 | 1.14 | -38.11 | N-ASC_02 | 0.02 | -28.96 |
| ASC_03 | 1.97 | -42.28 | N-ASC_03 | 0.15 | -66.63^*^ |
| ASC_04 | 2.16 | -81.70 | N-ASC_04 | 2.55 | 38.87^*^ |
| ASC_05 | 0.87 | -63.56 | N-ASC_05 | 5.02 | -275.56^*^ |
| ASC_06 | 0.79 | -193.39^*^ | N-ASC_06 | 0.65 | -22.37 |
| ^†^ASC_07 | 45.42^*^ | 59.83 | N-ASC_07 | 0.35 | -72.20^*^ |
| ^†^ASC_08 | 18.48^*^ | -560.05 | N-ASC_08 | 0.06 | 5.28 |
| ASC_09 | 0.43 | 25.00 | N-ASC_09 | 3.87 | -31.54^*^ |
| ASC_10 | 0.01 | -97.32^*^ | N-ASC_10 | 0.00 | 10.36 |
| ASC_11 | 8.39 | -120.13 | N-ASC_11 | 0.87 | 37.26 |
| ASC_12 | 0.08 | -11.42 | N-ASC_12 | 0.81 | 156.84^*^ |
| ASC_13 | 1.19 | -199.12^*^ | N-ASC_13 | 0.90 | -72.26^*^ |
| ASC_14 | 1.39 | -109.24^*^ | N-ASC_14 | 1.13 | -5.66^*^ |
| ASC_15 | 0.15 | 114.43^*^ | N-ASC_15 | 0.82 | -141.37 |
| ASC_16 | 0.60 | -109.47^*^ | N-ASC_16 | 1.75 | -20.13 |
| ASC_17 | 2.00 | 17.49^*^ | N-ASC_17 | 1.89 | -54.41^*^ |
| ASC_18 | 0.41 | -146.02^*^ | N-ASC_18 | 0.36 | 164.64^*^ |
| ASC_19 | 0.20 | -200.71^*^ | N-ASC_19 | 0.04 | 66.72^*^ |
| ASC_20 | 3.48 | -37.93 | N-ASC_20 | 0.34 | -96.40^*^ |

“^†^” indicates that the participant showed a difference in slope. “^*^” indicates a significant difference between execution and imagination tasks at the 0.01 level. A negative y-intercept indicates a higher y-intercept for imagination, while a positive y-intercept indicates a lower y-intercept for imagination.

1. Execution and Perception

The set of analyses comparing the regression lines for the MTs in the execution and perception tasks revealed that the regressions for the majority of the participants had slopes that did not statistically differ, and y-intercepts that were higher for execution than perception tasks. Specifically, the comparisons of the regression lines between the perception and execution MTs revealed that the slopes for the participants were not significantly different for 18 of the 20 participants in the autistic group and for 18 of the 20 participants in the non-autistic group. Of the 18 autistic participants whose slopes did not differ, 14 had higher y-intercepts for execution MTs than perception MTs and 4 had y-intercepts which did not differ between perception MTs and execution MTs. For the 18 participants in the non-autistic group whose slopes did not differ, 11 had higher y-intercepts for execution MTs than for perception MTs, 3 had lower y-intercepts for execution MTs than perception MTs, and 4 had y-intercepts which did not differ between perception MTs and execution MTs (see Table 6).

Table 6. Slope and y-intercept differences between execution and perception tasks

| Autistic group | Execution-Perception | | Non-autistic group | Execution-Perception | |
| --- | --- | --- | --- | --- | --- |
|  | Slope  (ms/ID) | y-intercept  (ms) |  | Slope (ms/ID) | y-intercept  (ms) |
| ASC_01 | 1.46 | 198.97^*^ | N-ASC_01 | 0.39 | 93.03^*^ |
| ASC_02 | 0.18 | 149.57^*^ | N-ASC_02 | 0.28 | 329.66^*^ |
| ASC_03 | 2.97 | 139.35^*^ | N-ASC_03 | 1.57 | -61.24^*^ |
| ^†^ASC_04 | 5.39^*^ | 366.96 | ^†^N-ASC_04 | 13.32^*^ | 303.58 |
| ASC_05 | 1.53 | 210.83^*^ | N-ASC_05 | 0.39 | 78.47^*^ |
| ASC_06 | 0.60 | 324.95^*^ | N-ASC_06 | 0.26 | 121.09^*^ |
| ASC_07 | 2.80 | 92.84 | N-ASC_07 | 0.22 | 200.88^*^ |
| ASC_08 | 1.34 | 50.76^*^ | N-ASC_08 | 0.03 | 64.90 |
| ASC_09 | 3.35 | 48.39 | N-ASC_09 | 0.10 | 61.28^*^ |
| ASC_10 | 0.06 | 91.83^*^ | N-ASC_10 | 1.13 | 128.71 |
| ASC_11 | 1.04 | 155.32^*^ | N-ASC_11 | 0.61 | 114.29^*^ |
| ASC_12 | 2.22 | 56.30 | N-ASC_12 | 0.00 | 80.77^*^ |
| ASC_13 | 0.05 | 85.22^*^ | N-ASC_13 | 0.72 | -44.41^*^ |
| ASC_14 | 1.83 | 18.25 | N-ASC_14 | 0.01 | 49.10 |
| ASC_15 | 1.08 | 219.13^*^ | N-ASC_15 | 0.54 | 328.47^*^ |
| ASC_16 | 1.73 | 97.22^*^ | ^†^N-ASC_16 | 6.29^*^ | 44.28 |
| ASC_17 | 3.96 | 51.73^*^ | N-ASC_17 | 0.82 | -123.98 |
| ASC_18 | 1.17 | 126.21^*^ | N-ASC_18 | 0.15 | 362.81^*^ |
| ASC_19 | 0.21 | 172.56^*^ | N-ASC_19 | 0.04 | 129.14^*^ |
| ^†^ASC_20 | 11.87^*^ | 131.64 | N-ASC_20 | 2.10 | -14.23^*^ |

“^†^” indicates that the participant showed a difference in slope. “^*^” indicates a significant difference between execution and perception task at the 0.01 level. A negative y-intercept indicates a higher y-intercept for perception, while a positive y-intercept indicates a lower y-intercept for perception.

1. Imagination and Perception

Finally, the set of analyses comparing the regression lines for the MTs in the imagination and perception tasks revealed that the regressions for the majority of the participants had slopes that did not statistically differ, and higher y-intercepts on the imagination task than on the perception task. Specifically, the comparisons of the regression lines between the imagination and perception MTs revealed that the slopes for the participants were not significantly different for 17 of the 20 participants in the autistic group and for all 19 of the 20 participants in the non-autistic group. Of the 17 autistic participants whose slopes did not differ, 14 had higher y-intercepts for imagination MTs than for perception MTs and 3 had y-intercepts which did not differ between perception MTs and imagination MTs. For the 19 participants in the non-autistic group whose slopes did not differ, 13 had higher y-intercepts for imagination MTs than for perception MTs and 6 had y-intercepts which did not differ between perception MTs and imagination MTs (see Table 7).

Table 7. Slope and y-intercept differences between imagination and perception tasks

| Autistic group | Imagination-Perception | | Non-autistic group | Imagination-Perception | |  |
| --- | --- | --- | --- | --- | --- | --- |
|  | Slope (ms/ID) | y-intercept  (ms) |  | Slope  (ms/ID) | y-intercept  (ms) |  |
| ASC_01 | 0.43 | 228.42^*^ | N-ASC_01 | 0.04 | 497.11^*^ |  |
| ASC_02 | 2.26 | 187.67^*^ | N-ASC_02 | 0.10 | 358.62^*^ |  |
| ASC_03 | 0.65 | 181.62^*^ | N-ASC_03 | 1.88 | 5.39^*^ |  |
| ASC_04 | 0.39 | 448.66^*^ | N-ASC_04 | 0.35 | 264.72^*^ |  |
| ASC_05 | 3.32 | 274.39^*^ | N-ASC_05 | 3.39 | 354.03^*^ |  |
| ASC_06 | 2.84 | 518.33^*^ | N-ASC_06 | 0.17 | 143.46^*^ |  |
| ASC_07 | 0.78 | 33.02^*^ | N-ASC_07 | 0.99 | 273.08^*^ |  |
| ^†^ASC_08 | 8.25^*^ | 610.81 | N-ASC_08 | 0.00 | 59.62 |  |
| ASC_09 | 1.81 | 23.39 | ^†^N-ASC_09 | 5.32^*^ | 92.82 |  |
| ASC_10 | 0.15 | 189.15^*^ | N-ASC_10 | 1.16 | 118.35 |  |
| ^†^ASC_11 | 11.17^*^ | 275.45 | N-ASC_11 | 4.64 | 77.03^*^ |  |
| ASC_12 | 1.82 | 67.72^*^ | N-ASC_12 | 0.55 | -76.07 |  |
| ASC_13 | 1.30 | 284.34^*^ | N-ASC_13 | 2.13 | 27.85 |  |
| ASC_14 | 0.10 | 127.50^*^ | N-ASC_14 | 0.62 | 54.77^*^ |  |
| ASC_15 | 0.73 | 104.71 | N-ASC_15 | 0.26 | 469.85^*^ |  |
| ASC_16 | 1.87 | 206.69^*^ | N-ASC_16 | 0.78 | 64.41^*^ |  |
| ASC_17 | 0.91 | 34.24 | N-ASC_17 | 2.00 | -69.57 |  |
| ASC_18 | 4.58 | 272.22^*^ | N-ASC_18 | 0.17 | 198.17^*^ |  |
| ASC_19 | 0.64 | 373.27^*^ | N-ASC_19 | 0.00 | 62.41^*^ |  |
| ^†^ASC_20 | 20.08^*^ | 169.57 | N-ASC_20 | 0.80 | 82.16 |  |

“^†^” indicates that the participant showed a difference in slope. “^*^” indicates a significant difference between imagination and perception task at the 0.01 level. A negative y-intercept indicates a higher y-intercept for perception, while a positive y-intercept indicates a lower y-intercept for perception.


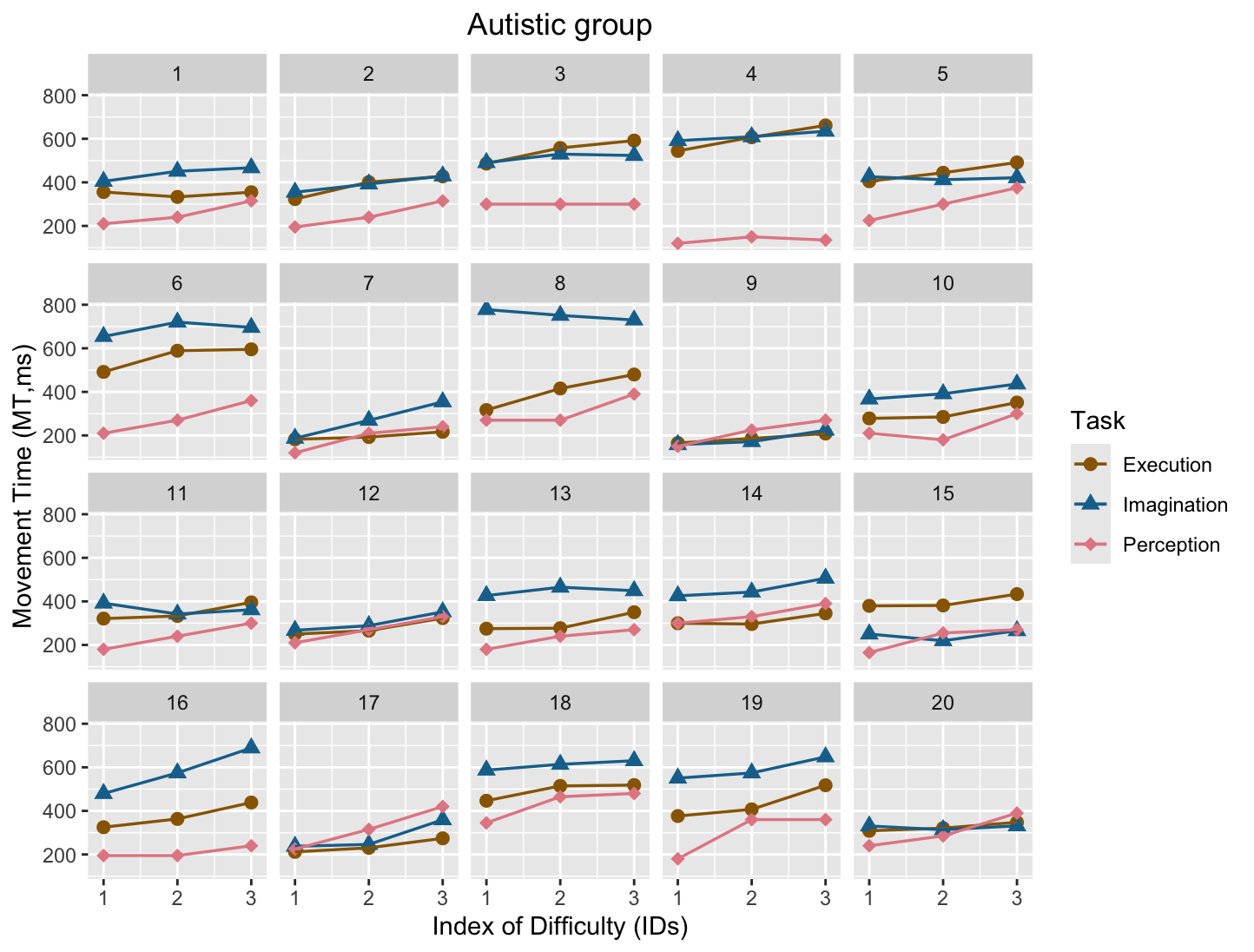


Fig 5.MT as a function of ID across Execution, Imagination, and Perception tasks for the autistic group. Each panel represents individual participants (Participant number from1 to 20), with MT plotted against increasing levels of ID (1, 2, and 3). The tasks are color-coded, with brown for the Execution task, blue for the Imagination task, and pink for the Perception task. Points represent the mean MT for each ID level, and lines connect these points to show the trend in performance across difficulty levels for each task.


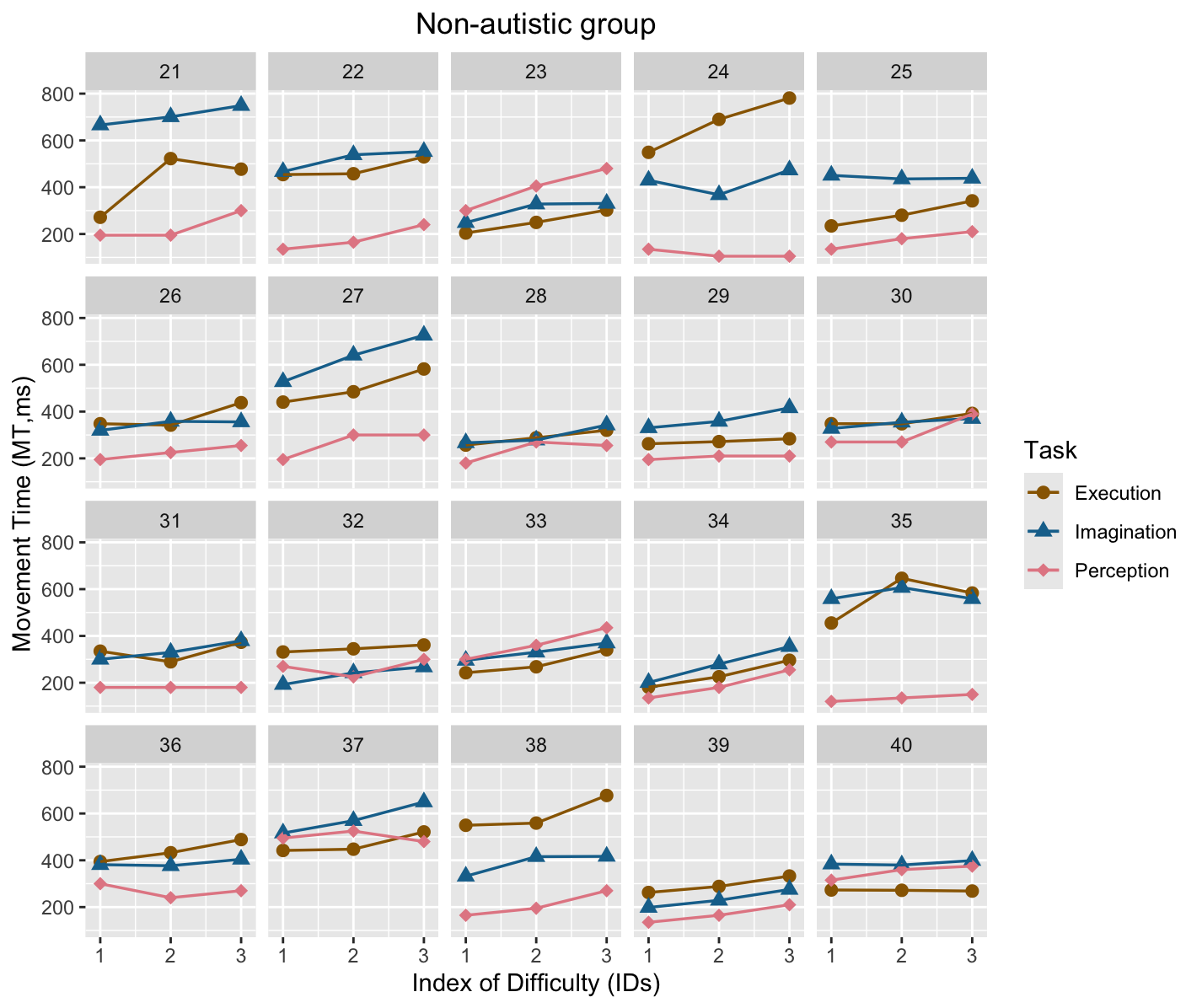


Fig 6. MT as a function of ID across Execution, Imagination, and Perception tasks for the non-autistic group. Each panel represents individual participants (Participant number from21 to 40), with MT plotted against increasing levels of ID (1, 2, and 3). The tasks are color-coded, with brown for the Execution task, blue for the Imagination task, and pink for the Perception task. Points represent the mean MT for each ID level, and lines connect these points to show the trend in performance across difficulty levels for each task.

## **Reference**

1. Wong, L., Manson, G. A., Tremblay, L. & Welsh, T. N. On the relationship between the execution, perception, and imagination of action. *Behav. Brain Res.* **257**, 242–252 (2013).
